# Electronic Health Interventions and Cervical Cancer Screening: Systematic Review and Meta-Analysis

**DOI:** 10.2196/58066

**Published:** 2024-10-31

**Authors:** Xiaoxia Liu, Lianzhen Ning, Wenqi Fan, Chanyi Jia, Lina Ge

**Affiliations:** 1 Department of Obstetrics and Gynecology Shengjing Hospital of China Medical University, Nanhu Campus Shenyang China; 2 Department of Nursing China Medical University Shenyang China

**Keywords:** cervical cancer, electronic health record, cancer screening, HPV, women's health, electronic health interventions

## Abstract

**Background:**

Cervical cancer is a significant cause of mortality in women. Although screening has reduced cervical cancer mortality, screening rates remain suboptimal. Electronic health interventions emerge as promising strategies to effectively tackle this issue.

**Objective:**

This systematic review and meta-analysis aimed to determine the effectiveness of electronic health interventions in cervical cancer screening.

**Methods:**

On December 29, 2023, we performed an extensive search for randomized controlled trials evaluating electronic health interventions to promote cervical cancer screening in adults. The search covered multiple databases, including MEDLINE, the Cochrane Central Registry of Controlled Trials, Embase, PsycINFO, PubMed, Scopus, Web of Science, and the Cumulative Index to Nursing and Allied Health Literature. These studies examined the effectiveness of electronic health interventions on cervical cancer screening. Studies published between 2013 and 2022 were included. Two independent reviewers evaluated the titles, abstracts, and full-text publications, also assessing the risk of bias using the Cochrane Risk of Bias 2 tool. Subgroup analysis was conducted based on subjects, intervention type, and economic level. The Mantel-Haenszel method was used within a random-effects model to pool the relative risk of participation in cervical cancer screening.

**Results:**

A screening of 713 records identified 14 articles (15 studies) with 23,102 participants, which were included in the final analysis. The intervention strategies used in these studies included short messaging services (4/14), multimode interventions (4/14), phone calls (2/14), web videos (3/14), and internet-based booking (1/14). The results indicated that electronic health interventions were more effective than control interventions for improving cervical cancer screening rates (relative risk [RR] 1.464, 95% CI 1.285-1.667; *P*<.001; *I* ^2^=84%), cervical cancer screening (intention-to-treat) (RR 1.382, 95% CI 1.214-1.574; *P*<.001; *I* ^2^=82%), and cervical cancer screening (per-protocol; RR 1.565, 95% CI 1.381-1.772; *P*<.001; *I* ^2^=74%). Subgroup analysis revealed that phone calls (RR 1.82, 95% CI 1.40-2.38), multimode (RR 1.62, 95% CI 1.26-2.08), SMS (RR 1.41, 95% CI 1.14-1.73), and video- and internet-based booking (RR 1.25, 95% CI 1.03-1.51) interventions were superior to usual care. In addition, electronic health interventions did not show a statistically significant improvement in cervical cancer screening rates among women with HPV (RR 1.17, 95% CI 0.95-1.45). Electronic health interventions had a greater impact on improving cervical cancer screening rates among women in low- and middle-income areas (RR 1.51, 95% CI 1.27-1.79). There were no indications of small study effects or publication bias.

**Conclusions:**

Electronic health interventions are recommended in cervical cancer screening programs due to their potential to increase participation rates. However, significant heterogeneity remained in this meta-analysis. Researchers should conduct large-scale studies focusing on the cost-effectiveness of these interventions.

**Trial Registration:**

CRD42024502884; https://www.crd.york.ac.uk/PROSPERO/display_record.php?RecordID=502884

## Introduction

Cervical cancer is a prevalent gynecologic malignancy within the female reproductive system. Based on the most recent data from the World Health Organization’s (WHO) International Agency for Research on Cancer in 2020, an estimated 6 million women worldwide are projected to be diagnosed with cervical cancer, accounting for 3.1% of all new cancer cases globally. In addition, about 3 million women are projected to die from the disease, representing 7.7% of all female deaths from cancer worldwide [1]. Cervical cancer develops gradually, often taking years or even decades to progress into a precancerous lesion, with clinical signs typically not apparent during the early stages of onset. Symptoms such as vaginal bleeding and discharge generally manifest in the intermediate to late stages, by which point the cancer may have invaded surrounding tissues and organs, surpassing the optimal window for treatment [2]. The WHO has initiated a worldwide effort to eliminate cervical cancer by promoting vaccination, screening, and treatment, given that the disease is preventable with current knowledge and technologies [3]. Therefore, cervical cancer screening is essential for the early detection of human papillomavirus (HPV) infection and cervical cytopathy, facilitating early intervention that can significantly reduce both the incidence and mortality rates of cervical cancer.

Over 1.5 billion women globally have never participated in cervical cancer screening [4]. Cancer screening begins by identifying and assessing eligible individuals, and it has been central to health care reform legislation and quality improvement initiatives. To optimize the benefits of screening, both population-based approaches and timely follow-up of abnormal test results are essential [5]. The primary methods for cervical cancer screening include Papanicolaou smears, HPV DNA testing, cytological examinations, and colposcopy. The WHO advocates HPV DNA testing as the preferred initial screening approach, regardless of whether a triage strategy is used. Women who test positive in the HPV DNA test are recommended to undergo further evaluation through genotyping, colposcopy, visual inspection with acetic acid, or cytological examination [6]. While many high-income countries have implemented screening programs, disparities persist among specific population subgroups [7]. Consequently, numerous studies have implemented interventions to enhance screening coverage among women.

Previously, 4 meta-analyses investigated interventions to boost the implementation and uptake of cervical cancer screening, with 2 reviews confirming the effectiveness of health education in improving screening rates. A review by Musa et al [8] demonstrated that cervical cancer education and screening reminders significantly increase screening rates, especially among women with lower educational levels. Brevik et al [9] showed that culturally tailored health education can improve cervical cancer screening rates among minority women. Research by Costa et al [10] confirmed that sending HPV self-testing kits directly to women is more effective at increasing cervical cancer screening rates than simply inviting women to screenings. Alam et al [7] conducted a systematic review of interventions aimed at improving cervical cancer screening among immigrant and refugee women, discovering that health education, screening guidance, and written information interventions effectively enhance screening rates. However, the scope and convenience of these measures are limited, and new strategies are needed to address this issue. The expanding use of electronic health technologies in medical informatics, public health, business, and emerging domains such as health care has broadened the applications of health services and information, which are provided or enhanced through the internet and associated technologies [11,12].

Electronic health allows for the delivery of health information in an accessible manner. During the COVID-19 pandemic, the use of electronic health platforms, such as websites and video conferencing, proliferated [13]. Numerous in-person appointments have been replaced by telehealth, which provides a scalable and adaptable means of offering support, monitoring patient-reported health outcomes, and facilitating continuity of care between hospital visits [14]. Significantly, patients find electronic health initiatives useful and acceptable, and through co-design, these initiatives can support patient-centered care [15]. Many high-income countries use electronic health interventions and media campaigns as educational strategies to promote cervical cancer screening among a broader population of women [16,17]. This approach might help achieve the WHO’s suggested elimination targets, which call for 90% of women with precancer and cancer to be treated or managed, 70% of women to be tested by the age of 30, and 90% of girls to receive the full HPV vaccination [3].

Examples of electronic health intervention technologies include web-based tactics, email, mobile or smartphone apps, text messaging, digital games, wearable or monitoring devices, and telemedicine or telehealth [18]. A systematic review by Romli et al [19] on the effectiveness of electronic health interventions for enhancing cervical cancer knowledge found that a mixed approach, including electronic health movies, video education, and didactic sessions, increased cervical cancer screening rates. This indicates that electronic health interventions for health education hold significant potential to improve cervical cancer screening rates. However, empirical research assessing the efficacy of electronic health interventions is needed to provide supporting evidence. A systematic evaluation of mobile technology for cervical cancer screening in low- and middle-income countries, published by Zhang et al [20], revealed that telephone reminders or SMSs significantly increased cervical cancer screening rates compared with traditional communication methods, such as postal mail. The study systematically examined the effects of mobile health intervention strategies on cervical cancer screening rates, specifically in relation to smartphones. No specific studies have reported the effectiveness of more comprehensive electronic health interventions in improving cervical cancer screening rates. Previous studies have been limited to specific groups or a single approach to electronic health intervention. However, the WHO advocates for a cervical cancer elimination program targeting the entire population [3]. This study encompasses global demographics and incorporates comprehensive electronic health interventions, including, but not limited to, text messages, video screens, apps, voice interactions, websites, and podcasts. Electronic health interventions constitute long-term interventions, which may lead to loss to follow-up, thereby impacting the assessment of their effectiveness. Previous meta-analyses did not further explore intention-to-treat (ITT) and per-protocol (PP) outcomes; therefore, this study extends the evaluation to include ITT and PP analyses of the effectiveness of electronic health interventions in cervical cancer screening.

We conducted a systematic review and meta-analysis to compare the effectiveness of comprehensive electronic health interventions with nonelectronic health interventions in terms of cervical cancer screening rates. In addition, we compared whether the impact of electronic health interventions on cervical cancer screening rates varied based on subjects, intervention type, and economic level. Comprehensive electronic health interventions are defined as all patient-oriented electronic health interventions, including, but not limited to, text messages, video screens, apps, voice interactions, websites, and podcasts. The results provide research evidence on the impact of electronic health interventions on cervical cancer screening rates, which may inform the design of strategies to improve global cervical cancer screening.

## Methods

### Study Design and Registration

This systematic review was registered with the PROSPERO (International Prospective Register of Systematic Reviews; CRD42024502884) and was conducted and reported in accordance with the PRISMA (Preferred Reporting Items for Systematic Reviews and Meta-Analyses) guidelines (checklist provided in Multimedia Appendix 1). In the course of the study, to clearly determine the effectiveness of electronic health interventions directly applied to patients and to ensure the accuracy and referability of the combined effect sizes after the meta-analysis, health care workers were excluded from the inclusion criteria, and only patient-related electronic health interventions were included.

### Inclusion and Exclusion Criteria

We included peer-reviewed articles published in English that met the eligibility criteria based on the PICOS (Population, Intervention, Comparison, Outcome, and Study Design) strategy (Textbox 1).

**Study inclusion and exclusion criteria defined by the Population, Intervention, Comparison, Outcome, and Study Design.**

**Inclusion criteria**

- Population: women aged 18 years or older receiving cervical cancer screening.
- Intervention: examples of patient-directed electronic health treatments include email, video conferences, videos, activity trackers, websites, podcasts, chat rooms, mobile applications, and text messages or SMS.
- Comparison: any comparator was acceptable, including a nonintervention group or an alternative group using nonsocial media and nonelectronic health interventions.
- Outcome: primary outcome indicator such as, predominantly reported cervical cancer screening rates in the included randomized controlled trials; secondary outcome indicators, including results of the intention-to-treat analysis and the per-protocol analysis of cervical cancer screening rates. The receipt of cervical cancer screening was defined as the completion of at least 1 cervical cancer screening test, including the Papanicolaou test, human papillomavirus DNA test, cytological examinations or colposcopy, from the start of the study to the end of follow-up. Both self-reported screening status (ie, patient reports) and provider-verified screening status (ie, medical records) were acceptable methods for assessing cervical cancer coverage.
- Study design: randomized controlled trials.

**Exclusion criteria**

- Population: studies focusing on the impact of electronic health interventions on health care workers and community workers to improve cervical cancer screening rates.
- Intervention: studies discussing how electronic health can be used to improve the accuracy of cervical cancer detection.
- Comparison: control group interventions that included electronic health components.
- Outcome: studies focused solely on knowledge, attitudes, or intentions regarding cervical cancer.
- Study design: editorials, letters, reviews, commentary pieces, and other nonresearch articles.

Only English-language publications were considered. When full-text papers were not available, the study authors were contacted to obtain the articles. If the eligibility requirements were met and data on cervical cancer screening were available, the abstract was included in the event that the authors did not reply. In addition, we excluded studies that focused on using electronic health to improve cancer screening rates across multiple types when cervical cancer screening data could not be isolated.

### Information Sources and Searches

A total of 8 electronic databases (from inception to present) were searched on December 29, 2023: MEDLINE, Cochrane Central Registry of Controlled Trials, Embase, PsycINFO, PubMed, Scopus, Web of Science, and the Cumulative Index to Nursing and Allied Health Literature. Authors LXX and NLZ conducted the search by combining terms indicative of cervical cancer screening (eg, cervical cancer, screen*, human papillomavirus DNA tests, and Papanicolaou test) with terms indicative of electronic health (eg, mobile phone, SMS, and videos). In addition, we checked the reference lists of the retrieved articles and previous meta-analyses and reviews for additional studies. The search queries used in this review are detailed in the Multimedia Appendix 2. Studies published between 2013 and 2022 were included.

### Study Selection

We exported the search results into a citation management system. After duplicates were eliminated, LXX (all articles), NLZ, and FWQ (each evaluated half) independently reviewed the titles and abstracts against the inclusion criteria. For full-text review, abstracts containing ambiguous information were included. LXX (18 articles), NLZ (17 articles), JCY (16 articles), FWQ (17 articles), and GLN (21 articles) reviewed full-text articles. LXX reviewed each article to ensure it was appropriately included or excluded.

### Data Collection Process

To extract the data, a prespecified electronic data extraction table was created using the Cochrane Collaboration’s Risk of Bias (RoB) tool and the PRISMA guidelines. The extracted data included the study details (author, year, country of origin, study design, application model, and sample size); participant details (medical history and demographics); duration of the intervention and follow-up; details of the intervention and control groups; primary outcomes; and Cochrane rule of reference measures. Authors LXX (8 articles) and NLZ (6 articles) extracted data from full text publications. LXX verified the accuracy of all the data.

### Risk of Bias

The RoB in the included studies was evaluated using the Cochrane Collaboration’s RoB tool [21]. The domains assessed were selection bias (sequence generation and allocation concealment), reporting bias (selective outcome reporting), attrition bias (incomplete outcome data), and performance or detection bias (blinding of participants, staff, and outcome assessors). For the criteria of low, unclear, and high RoB, the Cochrane Handbook for Systematic Reviews of Interventions was consulted for both within-trial and across-trial assessments. Independent reviews of RoB were conducted by authors LXX (8 publications) and NLZ (6 papers). LXX reviewed each RoB evaluation to ensure accuracy. Publication bias was assessed by visually inspecting a funnel plot for the main outcome and conducting the Egger test [22]. To estimate the pooled effect while accounting for missing studies, the Duval and Tweedie trim-and-fill analysis was used [23].

### Statistical Methods

A descriptive presentation of the study, participant, and intervention features, as well as the RoB assessments, is provided. The relevant outcomes are presented in terms of absolute and relative values, and a random-effects model was used to aggregate relative risk (RR) by applying the Mantel-Haenszel technique. The study used data from the longest follow-up period if outcomes were evaluated at different time points. In cases where studies included multiple intervention arms, the meta-analysis included only the most complex intervention, defined as having the most components. The *I*^2^ statistic was used to compute statistical heterogeneity, with a cutoff value of ≥75% considered significant. The Cochran Q test (*P*<.1) was used to conduct a formal test of homogeneity, which is considered statistically significant [24]. Subgroup analysis was performed according to subjects (women with and without cervical cancer screening experience, women with a long absence from screening, and women with HPV), intervention type (phone call, SMS, multimode, web video, and internet-based booking), and economic level (high-income and middle- and low-income). This study used 2 types of sensitivity analyses: the leave-one-out method and the removal of highly biased studies. For statistical significance, a 2-tailed *P*<.05 was used. Meta-analyses were conducted using Stata (version 16.0, StataCorp LLC).

## Results

### Study Inclusion

**Figure 1:**
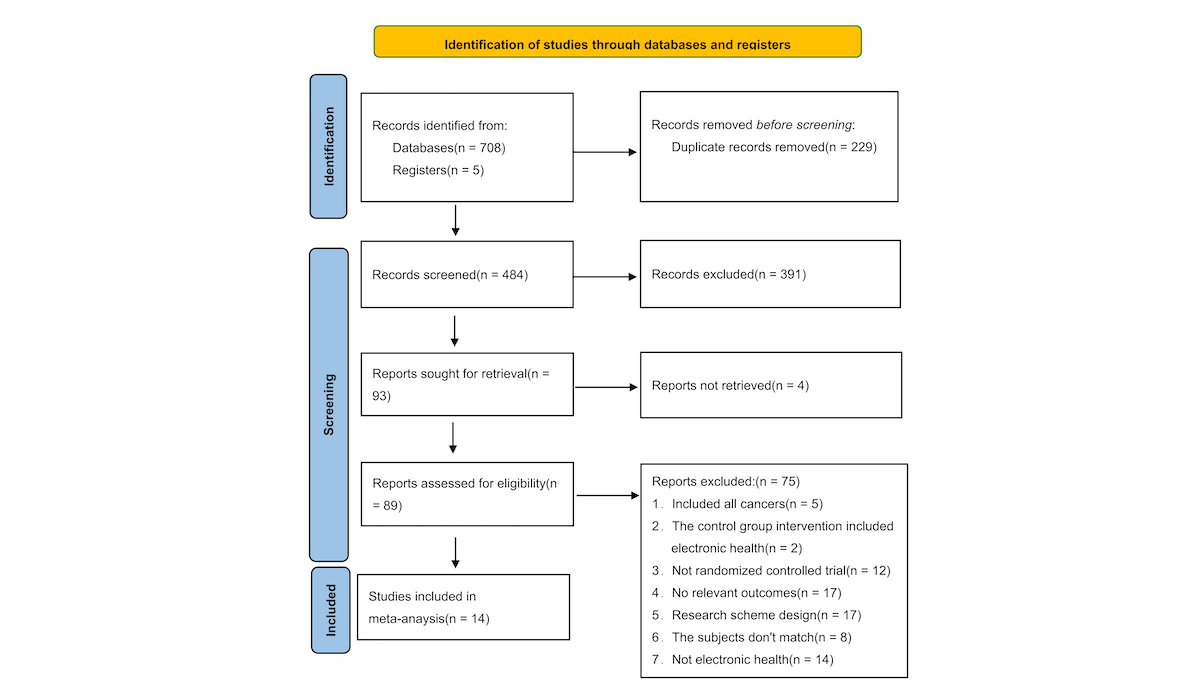
PRISMA (Preferred Reporting Items for Systematic Reviews and Meta-Analyses) flowchart of the study selection process.

After removing duplicates, the screening identified 14 articles (comprising 15 studies), [25-38], of which 11 [25-27,29-32,34-37] contained ITT results, and 12 [25-33,35,36,38] contained PP results (Figure 1), representing 23,102 unique patients (Table S1 in Multimedia Appendix 3). A total of 7 studies [27,28,31-33,35,36] had high RoB.

### Study Characteristics

The included studies were conducted in America, Europe, Asia, and Africa, and they were published between 2013 and 2022. Single-intervention studies made up the majority of the studies (10/14, 71%) [25-27,29,30,32-35,38] while multimode intervention studies accounted for 29% (4/14) [28,31,36,37]. The interventions were conducted by university-based research teams, governmental or commercial screening initiatives, or health care facilities or departments. The intervention strategies used in these studies included SMS in 4 studies [27,30,32,35], multimode interventions in 4 studies [28,31,36,37], phone call interventions in 2 studies [25,26], web videos in 3 studies [29,33,38], and internet-based booking in 1 study [34]. Outcomes were measured at several time points, including 2, 3, 4, 5, 6, 7, 12, and 14 months. The interventions in most studies (8/14) were based on theoretical models, including the Transtheoretical Model [26-30,32,36,38], theory of planned behavior [27], Health Belief Model [28-30], MINDSPACE (Messenger, Incentives, Norms, Defaults, Salience, Priming, Affect, Commitments, and Ego) framework [32], and social cognitive theory [29,36,38] (Table S1 in Multimedia Appendix 3).

There was a wide variation among the study participants. For example, in some studies, participants were targeted based on geographical region or by their profession as teachers [26,33], emergency patients [27], or young women [32,34]. Some of the participants were women from the general population (regardless of their participation in cervical cancer screening), some were women who had not participated in screening for a long time or had never participated in screening [26,27,37,38], and some were women with HPV [28,35]. Supplementary information regarding the interventions in the experimental and control groups is shown in Table S1 in Multimedia Appendix 3.

### Quality Assessment

**Figure 2:**
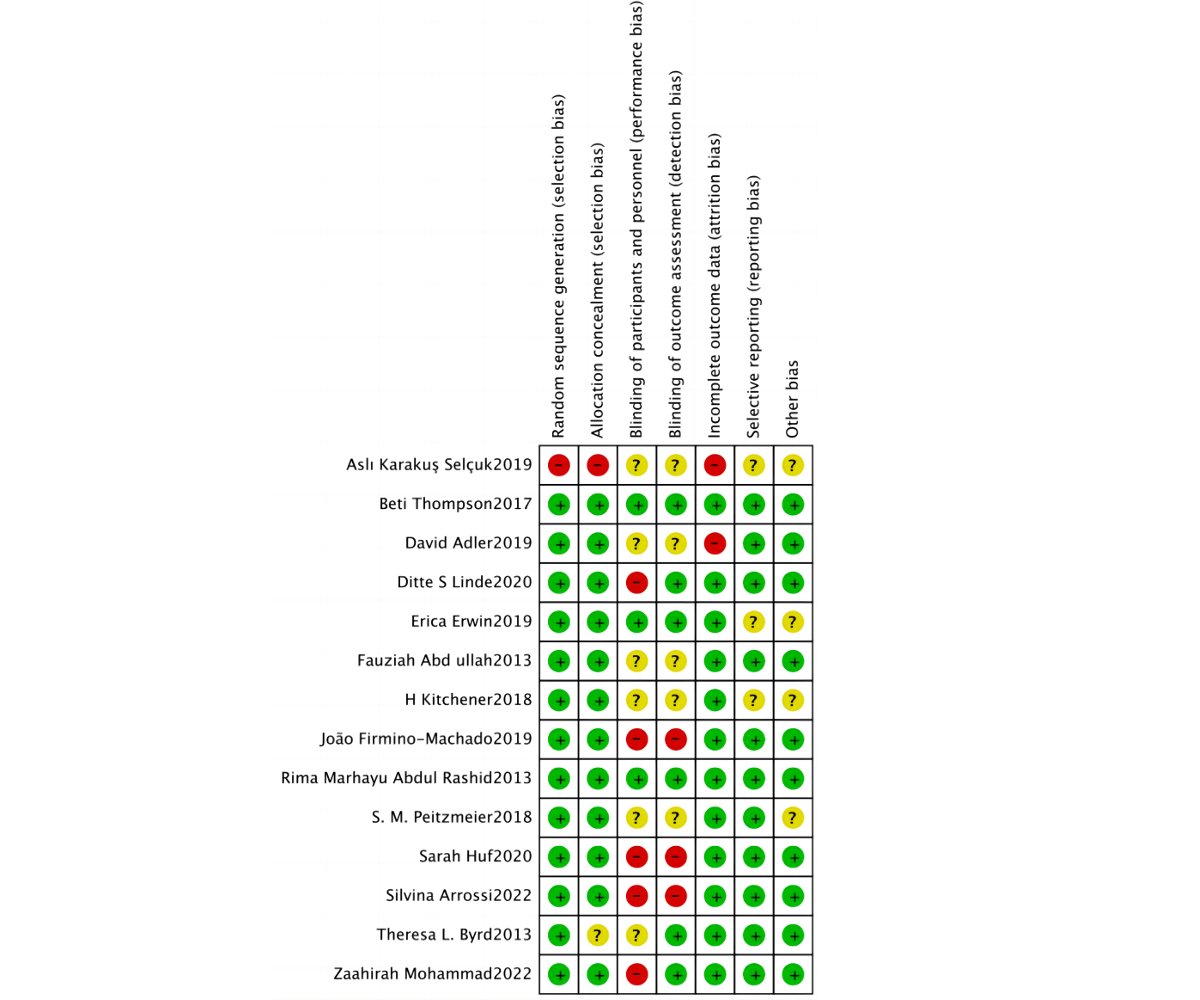
Risk of bias summary for the included randomized controlled trials (N=14) [25-38].

The RoB evaluations for the research included in the analysis are presented in Figures 2 [25-38] and 3. In summary, 50% (7/14) [27,28,31-33,35,36] of the randomized controlled trials (RCTs) included in the study were categorized as high risk, and the remaining RCTs (7/14) [26,27,29,30,33,34,37] were considered as having some concerns. Only 2 studies (2/14, 14%) [25,38] were classified as low risk. Studies were categorized as high risk due to issues in multiple areas, such as participant and staff blinding, outcome assessment blinding, and assignment effect blinding. Upon visual examination of the funnel plot (Figure 4), no indications of publication bias were observed. The effect sizes exhibited a relatively symmetrical distribution. Most effect sizes fell within the funnel plot, and those outside it were symmetrically distributed. Egger's regression intercept test yielded a significant result (Intercept=2.5, 95% CI 0.32-4.45; *P*=.03), indicating that the observed results might be influenced by publication bias. The trim and fill approach developed by Duval and Tweedie was used to evaluate the presence of publication bias. After 2 iterations of the analysis, no additional trials were deemed necessary, and the combined effect size remained consistent. After accounting for potential publication bias, the effect of the intervention remained statistically significant (*P*<.001). Although the observed results might have been influenced by publication bias, there was no indication of a small-study effect (Egger test: *t*=2.5; *P*=.03; 95% CI 0.32-4.45).

**Figure 3:**
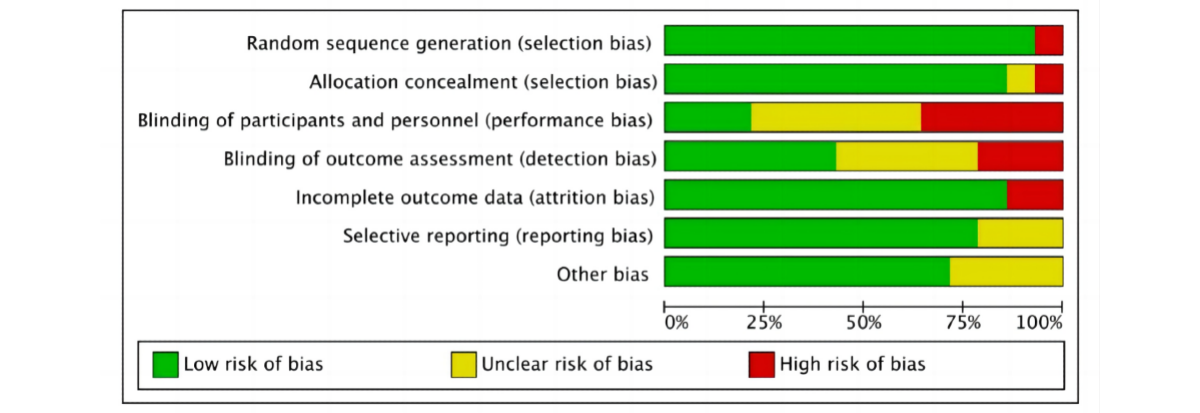
Analysis of overall risk of bias in the included randomized controlled trials (n=14).

**Figure 4:**
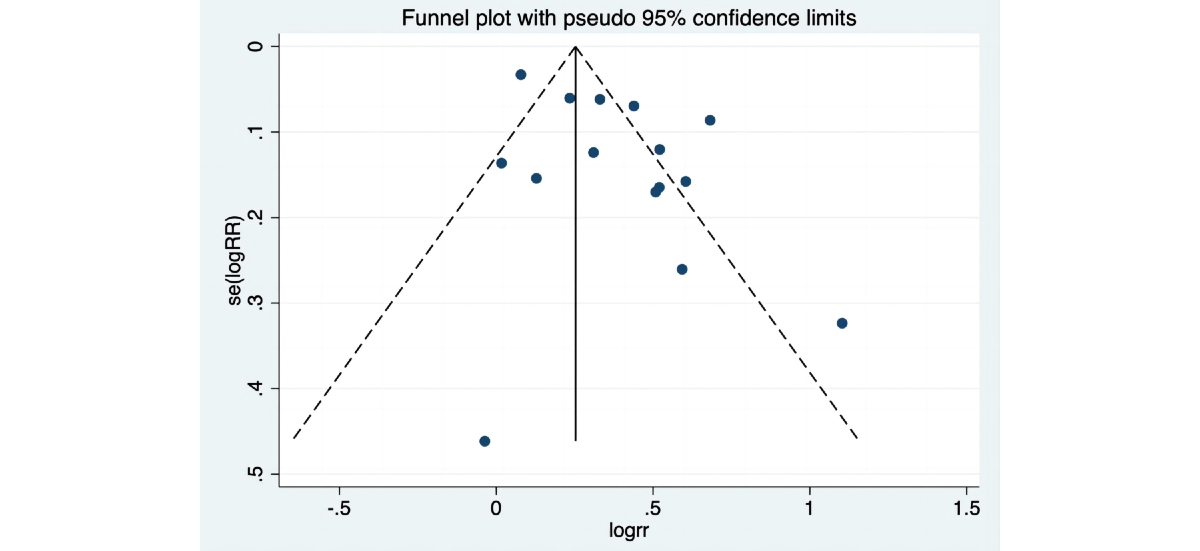
Funnel plot comparing electronic health interventions to nonelectronic health interventions in randomized controlled trials reporting on cervical cancer screening. RR: relative risk.

### Cervical Cancer Screening

**Figure 5:**
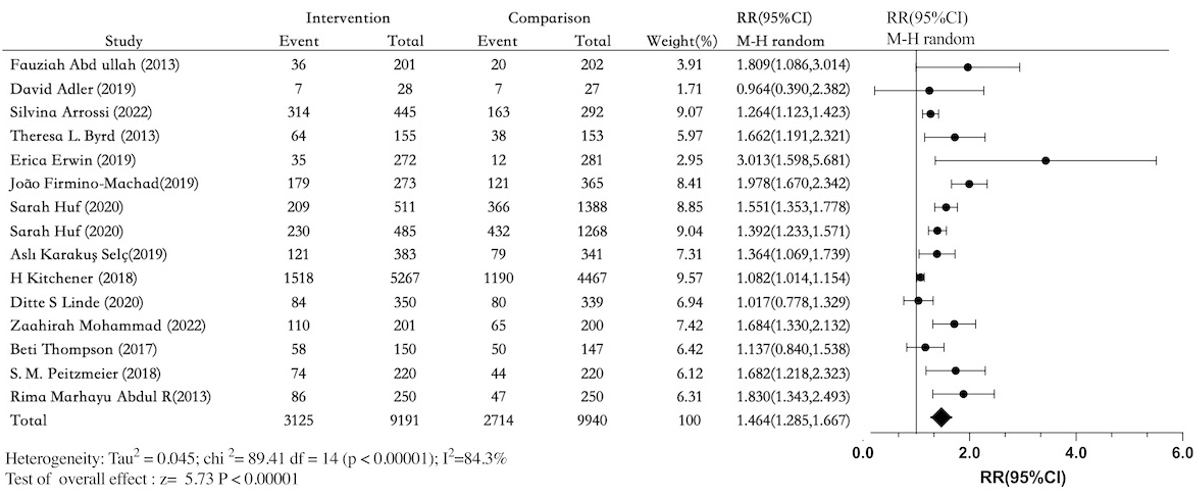
Forest plot comparing electronic health interventions with nonelectronic health interventions in randomized controlled trials for cervical cancer screening participation (n=14). RR: relative risk [25-38].

The cervical cancer screening rate was the primary outcome in 12 out of 14 articles (86%) [26-32,34-38]. In total, 10 studies [25,26,28-32,36-38] found that the intervention significantly increased cervical cancer screening rates. The intervention had an absolute impact of 34% (3125/9191) in the intervention arms. Between the intervention and comparison arms, there was an absolute risk difference of 11.9% (95% CI, 7.4-16.4), with 27% (2714/9940) screened in the comparison arm. Among the included RCTs, the overall pooled RR for participation in cancer screening was 1.464 (95% CI 1.285-1.667; Figure 5 [25-38]), meaning that receiving an electronic health intervention raised the odds of screening by 46%. Nonetheless, significant heterogeneity (*I*^2^=84%) was noted.

Similar effect estimates were obtained after stratification according to the intervention method. The order of magnitude of the effects of the various intervention modalities was as follows: phone call (RR 1.82, 95% CI 1.40-2.38; *I*^2^=0%), multimode (RR 1.62, 95% CI 1.26-2.08; *I*^2^=85%), SMS (RR 1.41, 95% CI 1.14-1.73; *I*^2^=71%), and web videos and internet-based booking (RR 1.25, 95% CI 1.03-1.51; *I*^2^=67%; Figure S3 in Multimedia Appendix 3). Subgroup analysis based on the intervention group revealed no significant difference in cervical cancer screening rates between women with HPV in the electronic health interventions group and the nonelectronic health interventions group (RR 1.17, 95% CI 0.95-1.45; *I*^2^=57%). Women with and those without cervical cancer screening experience (RR 1.56, 95% CI 1.32-1.90; *I*^2^=90%) and women with a long absence from screening (RR 1.41, 95% CI 1.08-1.85; *I*^2^=37%) showed a difference between the electronic health interventions group and the control group (Figure S4 in Multimedia Appendix 3). Subgroup analysis according to economic status showed a significant difference for the middle- and low-income group between the electronic health intervention group and the control group (RR 1.51, 95% CI 1.27-1.79; *I*^2^=68%). A significant difference was observed between the intervention and control groups in high-income countries. The RR was 1.41, with a 95% CI of 1.15-1.73, and the heterogeneity (*I*^2^) was 91%. Refer to Figure S5 in Multimedia Appendix 3 for further details. The sensitivity analysis demonstrated that the combined RR and *I*^2^ value were consistent (RR 1.46, 95% CI 1.29-1.66; refer to Figure S6 in Multimedia Appendix 3). After removing studies with a high risk of bias, a meta-analysis (n=7) compared the intervention and control groups at the end of the intervention. The analysis showed an RR of 1.56 (95% CI 1.21-2.02) for cervical cancer screening (refer to Figure S7 in Multimedia Appendix 3).

### Intention-To-Treat of Cervical Cancer Screening

**Figure 6:**
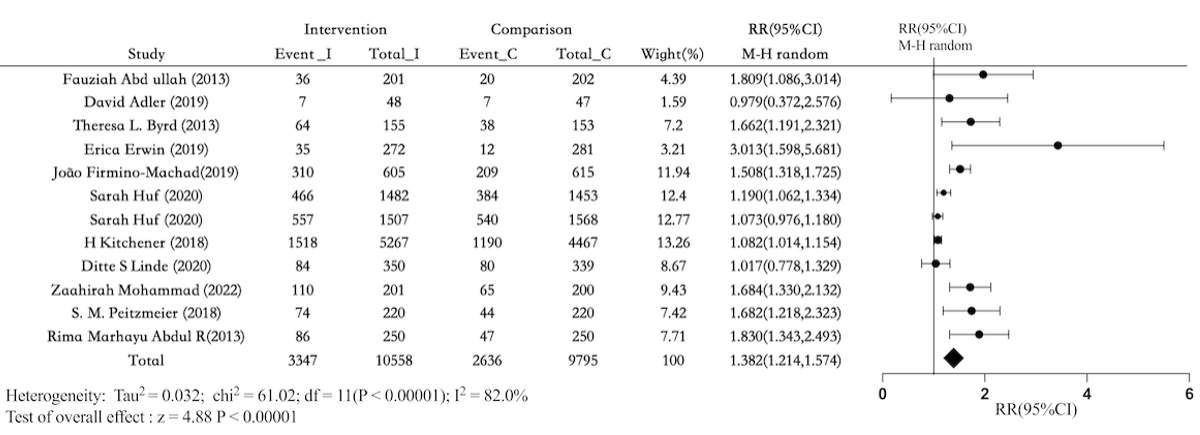
Forest plot comparing electronic health interventions with nonelectronic health interventions in randomized controlled trials for intention-to-treat analysis of cervical cancer screening participation (n=11). RR: relative risk [25-27,29,30-32,34-37].

A total of 11 studies [25-27,29-32,34-37] reported the results of ITT analysis of cervical cancer screening rates. The results indicated that electronic health can improve the cervical cancer screening rate of participants more effectively than that of the control group (RR 1.382, 95% CI 1.214-1.574; *P*<.001; *I*^2^=82.0%; Figure 6 [25-27,29-32,34-37]). The percentage of people screened in the comparison groups was 26.9% (2636/9795), while the absolute effect of screening in the intervention groups was 31.7% (3347/10558). The sensitivity analysis of the leave-one-out method, demonstrated that excluding any single study from the random-effects model did not result in a loss of significance (Figure S8 in Multimedia Appendix 3). The results of sensitivity analysis after excluding studies with high RoB show that the overall pooled RR were robust (RR 1.67, 95% CI 1.22-2.28; Figure S9 in Multimedia Appendix 3).

### PP of Cervical Cancer Screening

**Figure 7:**
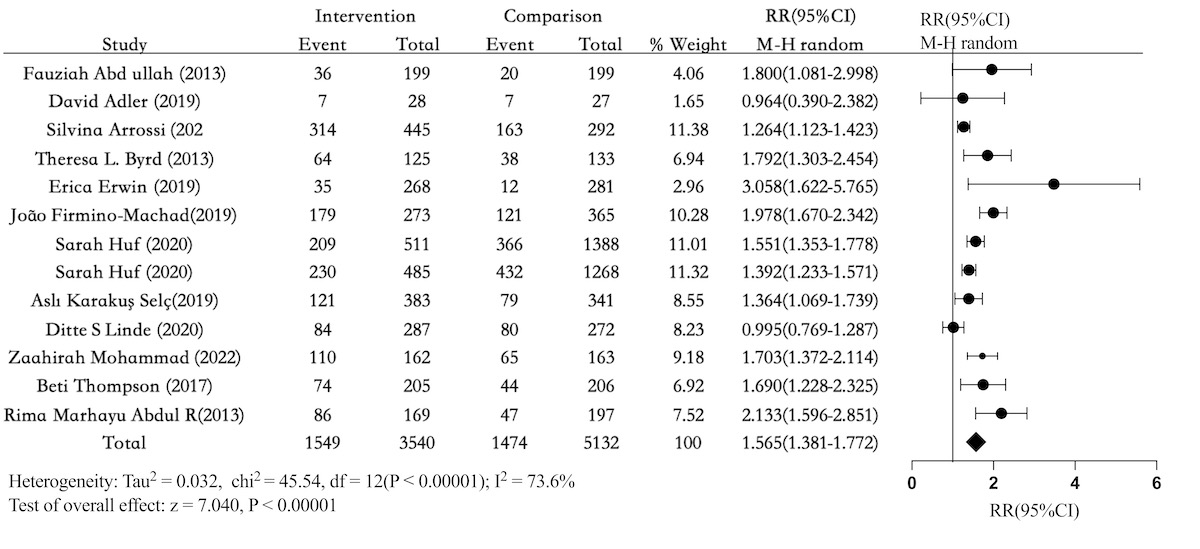
Forest plot comparing electronic health interventions with nonelectronic health interventions in randomized controlled trials for per-protocol analysis of cervical cancer screening participation (n=12). RR: relative risk [25-33,35,36,38].

The findings of the PP analysis of cervical cancer screening rates were published in 12 publications [25-33,35,36,38]. According to the findings, the total pooled effect estimate for PP of cervical cancer screening was 1.565 (95% CI 1.381-1.772, *I*^2^=74%; Figure 7 [25-33,35,36,38]). The intervention and comparison groups had an absolute risk difference of 15% (95% CI 10.2-19.8). Comparatively, the intervention group’s cancer screening rate was 44% (1549/3540), while the control group’s rate was 29% (1474/5132). The sensitivity analysis of the leave-one-out method, demonstrated that excluding any single study from the random-effects model did not result in a loss of significance (Figure S10 in Multimedia Appendix 3). After removing studies with a high RoB (n=5), a sensitivity analysis revealed a pooled RR value of 1.93 (95% CI 1.64-2.27; Figure S11 in Multimedia Appendix 3).

## Discussion

### Principal Findings

This meta-analysis evaluated the effectiveness of electronic health interventions in increasing cervical cancer screening rates. We found that electronic health interventions can significantly improve cervical cancer screening rates among women. The ITT and PP analyses also showed similar results.

Our systematic review included 14 papers (15 studies) that examined the impact of electronic health interventions on enhancing participation in cervical cancer screening. The overall effect size for cervical cancer screening rates significantly favored the electronic health interventions group (RR 1.464, 95% CI 1.285-1.667). In addition, the subgroup analysis revealed no discernible impact of electronic health interventions on increasing cervical cancer screening rates among women who tested positive for HPV. For individuals who qualified for a PP analysis, the effect size on cervical cancer screening rates was the largest (RR 1.565, 95% CI 1.381-1.772). The sensitivity analyses demonstrated the stability of the result estimates, indicating that electronic health interventions are effective in enhancing cervical cancer screening rates.

Three studies similar to the present study were published before 2021. Uy et al [39] and Ruco et al [12] evaluated the effectiveness of electronic health-related interventions for improving cancer screening rates. These studies included different types of cancer. Uy et al [39] focused on SMS interventions, and the results showed that SMS can improve the screening rates for cervical cancer and breast cancer. The main intervention studied by Ruco et al [12] was social media, and the results showed that mobile health technology, especially social media, can effectively improve cancer screening rates. However, the study by Ruco et al [12] included controlled clinical trials, whereas the present analysis included only RCTs, which may provide a more accurate pooled effect size. Tamuzi et al [40] looked at mobile health interventions for cervical cancer screening and found 17 papers in total, which were then categorized by type of intervention into a meta-analysis. In this study, the intervention is electronic health, which includes SMS, phone calls, multimode interventions, web videos, and internet-based booking. In their study, the intervention was evaluated in the context of mobile health, which included phone calls, letters, and text alerts. According to Tamuzi et al [40], the only intervention with statistically significant estimates of aggregate impacts was phone alerts. However, a meta-analysis of these treatments could not be carried out since only 1 article in their review discussed the effectiveness of SMS alerts. According to the current analysis, 4 out of the 14 publications included discussed how text messaging increased cervical cancer screening rates. The results of this study also suggest that text messaging is effective for improving cervical cancer screening rates (RR 1.41, 95% CI 1.14-1.73). In addition, his study was conducted in 2017, and most of the articles included in this study were published after 2017 (10/14). Our review offers a thorough and updated overview of this subject. In addition, this study analyzes the effect size of electronic health interventions on cervical cancer screening rates using PP and ITT analyses to clarify their effectiveness and extensibility.

The popularity of electronic health is increasing in parallel with technological advances [41]. In a survey of US adults, over 80% of individuals aged 18-49 years old and 73% of those aged 50-64 years old use social media sites [42]. Electronic health interventions include various methods, such as SMS, phone calls, and web videos [43]. The results of this study show that, regardless of the type of intervention, electronic health can improve cervical cancer screening rates. The results of this study show that the aggregate effect size was statistically significant for both ITT and PP analyses, which strongly supports the effectiveness of electronic health in improving cervical cancer screening rates.

When the studies were grouped according to the intervention method, the results showed that each of the intervention modalities (including phone calls, SMS, multimode, web videos, and internet-based booking) could increase the cervical cancer screening rate, but the combined effect size was highest when phone calls were used as the means of intervention (RR 1.82, 95% CI 1.40-2.38; *I*^2^=0%) [27,33]. Phone calls are a cost-effective intervention [44], because direct contact with the recipient allows timely responses to questions, which further validates the importance of direct communication [45]. However, phone calls intervention is associated with higher manpower costs. The development of voice interaction can save manpower costs while retaining some of the advantages of telephone intervention [46]. Further studies are necessary to understand the impact of voice interaction on cervical cancer screening rates.

When the studies were grouped according to economic level, the subgroup analysis results showed that the aggregate effect size of electronic health on cervical cancer screening rates was higher in low- and middle-income areas than in high-income areas, suggesting that electronic health is more effective in low- and middle-income populations. One possible reason for this result is that resources are scarce in low- and middle-income areas. Electronic health interventions can help the residents of low- and middle-income areas improve their awareness and attention to cervical cancer through internet-based health education, health knowledge dissemination, internet-based reminder services, and other forms, which may increase the motivation for screening [20,47]. In high-income areas, electronic health applications are more common, and electronic health incentives are therefore weaker for the high-income population than for the low-income population [48].

Subgroup analysis based on distinct intervention populations revealed no statistically significant differences in cervical cancer screening rates among women with HPV between the electronic health intervention group and the conventional intervention group. Most of the studies included in the analysis were based on the theoretical health belief model [28,30], and the main strategy was to provide patients with health education related to cervical cancer screening in the form of electronic health [26-28,35-37]. Overall, the screening rate in women with HPV (45%) was higher than the average rate (30.52%), possibly because women with HPV are more aware of their risk of cervical cancer as carriers of high-risk HPV [49,50]. Factors such as physician recommendations, secondary tests, and education also play a role in promoting their participation in cervical cancer screening, resulting in a relatively high screening rate. Because the main limitation to follow-up screening in women with HPV is not cervical cancer awareness, electronic health is not effective in improving screening rates in this population [51]. However, advances in science and technology are increasing the diversity of electronic health methods available, and the intervention mechanism is not limited to reminder education [52]. Further studies are needed to explore the effect of electronic health on cervical cancer screening rates in women with HPV.

The Egger’s test results of this study suggest potential publication bias; however, after using the trim and fill method, no additional studies were deemed necessary, and the combined effect size and *P* values remained unchanged, thereby affirming the robustness of these findings. The RoB results indicated that 50% of the studies exhibited high bias risk, prompting a sensitivity analysis excluding these high-risk studies, which enhanced the precision and reliability of the outcomes. After excluding studies with high bias risk, the pooled results were as follows: cervical cancer screening: 1.56 (95% CI 1.21-2.02), cervical cancer screening (ITT): 1.67 (95% CI 1.22-2.28), cervical cancer screening (PP): 1.93 (95% CI 1.64-2.27). Consequently, although studies with high bias risk may reduce some intervention effects, the overall effect size exhibited minimal variation, thereby confirming the robustness of the study results.

### Limitations

Despite the consistency of the pooled effect estimate in the subgroup and sensitivity analyses, significant heterogeneity remained in this meta-analysis. The variation in demographics, treatments, or outcome measures among the studies may be the cause of this. For instance, all adults aged 65 years or younger or highly specialized populations like ER patients or individuals with HPV were among the populations randomized in the studies included in this review. Furthermore, we only included papers published in English, which could have an impact on the meta-analysis’s findings. Despite our best efforts, we were unable to locate some of the records that the search identified. Finally, because it was unable to identify the outcomes unique to cervical cancer, RCTs that enrolled patients with a variety of malignancies and reported their combined findings were excluded. Furthermore, non-RCT designs (such as adaptive trials) were disregarded.

### Conclusion

In conclusion, the results of this study suggest that electronic health interventions may significantly impact increasing cervical cancer screening rates. However, the study exhibited a high degree of heterogeneity. Phone call–based electronic health interventions demonstrated the most substantial improvements in increasing cervical cancer screening rates, with low heterogeneity. Multimodal interventions constitute a growing segment of electronic health treatments. The RoB review highlighted variations in blinding processes, and only a few studies disclosed plans to upscale, assess the cost of the intervention, or maintain the outcomes. Future cost-effectiveness analyses should be conducted to further elucidate the scalability of electronic health interventions.

## Appendix

Multimedia Appendix 1 PRISMA (Preferred Reporting Items for Systematic Reviews and Meta-Analyses) checklist for the full text and abstract sections.

Multimedia Appendix 2 Search strategies of MEDLINE, Cochrane Central Registry of Controlled Trials, Embase, PsycINFO, PubMed, Scopus, Web of Science, and the Cumulative Index to Nursing and Allied Health Literature.

Multimedia Appendix 3 Supplementary material for this study, including general information on included studies, funnel plot results, subgroup analysis results, sensitivity analysis results.

## Abbreviations

HPV: human papillomavirus
ITT: intention-to-treat
MINDSCAPE: Messenger, Incentives, Norms, Defaults, Salience, Priming, Affect, Commitments, and Ego framework
PICOS: Population, Intervention, Comparison, Outcome, and Study design
PP: per-protocol
PRISMA: Preferred Reporting Items for Systematic Reviews and Meta-Analyses
PROSPERO: International Prospective Register of Systematic Reviews
RCT: randomized controlled trial
RoB: risk of bias
RR: relative risk
WHO: World Health Organization

## Data Availability

The datasets generated during or analyzed during this study are available from the corresponding author on reasonable request.
