# Supplementary material for: Electronic Health Interventions and Cervical Cancer Screening: Systematic Review and Meta-Analysis
Source: J Med Internet Res. 2024 Oct 31;26:e58066. doi: 10.2196/58066 (PMC11565089; doi:10.2196/58066)

Pubmed

((((((Uterine Cervical Neoplasms [MeSH Terms] OR Uterine Cervical Dysplasia [MeSH Terms] OR Cervical Intraepithelial Neoplasia [MeSH Terms] OR ((cervix [tw] OR cervical [tw] OR cervico* [tw]) AND (cancer* [tw] OR carcinoma OR adenocarcinoma OR neoplas* [tw] OR dysplas* [tw] OR dyskaryos* [tw] OR squamous [tw] OR CIN [tw] OR CINII* [tw] OR CIN2* [tw] OR CINIII* [tw] OR CIN3* [tw] OR SIL [tw] OR HSIL [tw] OR H-SIL [tw] OR LSIL [tw] OR L-SIL [tw] OR ASCUS [tw] OR AS-CUS [tw] )))) AND ((((Early Detection of Cancer[MeSH Terms]) OR (Early Detection of Cancer[Title/Abstract])) OR (Screening[Title/Abstract])) OR (screen[Title/Abstract]))) OR (("Human Papillomavirus DNA Tests"[Mesh]) OR ((((Human papillomavirus test[Title/Abstract]) OR (HPV DNA Test*[Title/Abstract])) OR (Human Papillomavirus DNA Test*[Title/Abstract])) OR (alphapapillomavirus[Title/Abstract])))) OR (("Papanicolaou Test"[Mesh]) OR ((((Pap smear[Title/Abstract]) OR (Papanicolaou Smear[Title/Abstract])) OR (Pap test*[Title/Abstract])) OR (apanicolaou Test*[Title/Abstract])))) AND (((((((((((((((((((mobile phone[Title/Abstract]) OR (smartphone[Title/Abstract])) OR (text-message[Title/Abstract])) OR (electronic-message[Title/Abstract])) OR (multimedia-message[Title/Abstract])) OR (electronic mail[Title/Abstract])) OR (phone applications[Title/Abstract])) OR (computer[Title/Abstract])) OR (podcast[Title/Abstract])) OR (videos[Title/Abstract])) OR (internet[Title/Abstract])) OR (website[Title/Abstract])) OR (chatroom[Title/Abstract])) OR (message board[Title/Abstract])) OR (activity tracker[Title/Abstract])) OR (electronic health[Title/Abstract])) OR (mobile health[Title/Abstract])) OR (telemedicine[Title/Abstract])) OR (electronic learning[Title/Abstract]))) AND ((RCT[Title/Abstract]) OR (Randomized controlled trial[Title/Abstract]))

Web of Science

| # | Search Query | Results |
| --- | --- | --- |
| 1 | TS=(cancer*) OR TS=(carcinoma) OR TS=(adenocarcinoma) OR TS=(neoplas* ) OR TS=(dysplas* ) OR TS=(dyskaryos* ) OR TS=(squamous) OR TS=( CIN ) OR TS=(CINII* ) OR TS=(CIN2* ) OR TS=(CINIII* ) OR TS=(CIN3* ) OR TS=(SIL) OR TS=(HSIL) OR TS=(H-SIL) OR TS=( L-SIL) OR TS=(LSIL) OR TS=(ASCUS) OR TS=(AS-CUS) and Preprint Citation Index (Exclude – Database) | 7339275 |
| 2 | (((TS=(cervix)) OR TS=(cervical ))) OR TS=(cervico*) and Preprint Citation Index (Exclude – Database) | 534857 |
| 3 | #1 AND #2 and Preprint Citation Index (Exclude – Database) | 298929 |
| 4 | ((TS=(Screening)) OR TS=(Screen*)) OR TS=(Early Detection of Cancer) and Preprint Citation Index (Exclude – Database) | 1817482 |
| 5 | #3 AND #4 and Preprint Citation Index (Exclude – Database) | 45517 |
| 6 | TS=(Human papillomavirus test) OR TS=(HPV DNA Test*) OR TS=(Human Papillomavirus DNA Test*) OR TS=(alphapapillomavirus) OR TS=(Papanicolaou Test) OR TS=(Pap smear) OR TS=(Papanicolaou Smear) OR TS=(Pap test) and Preprint Citation Index (Exclude – Database) | 50761 |
| 7 | #6 OR #5 and Preprint Citation Index (Exclude – Database) | 79766 |
| 8 | TS=(mobile phone) OR TS=(smartphone) OR TS=(text-message) OR TS=(electronic-message) OR TS=(multimedia-message) OR TS=(electronic mail) OR TS=(phone applications) OR TS=(computer) OR TS=(podcast) OR TS=(videos) OR TS=(internet) OR TS=(website) OR TS=(chatroom) OR TS=(message board) OR TS=(activity tracker) OR TS=(electronic health) OR TS=(mobile health) OR TS=(telemedicine) OR TS=(electronic learning) and Preprint Citation Index (Exclude – Database) | 5608461 |
| 9 | #8 AND #7 and Preprint Citation Index (Exclude – Database) | 7496 |
| 10 | (AB=(RCT)) OR AB=(Randomized controlled trial) and Preprint Citation Index (Exclude – Database) | 416040 |
| 11 | #10 AND #9 and Preprint Citation Index (Exclude – Database) | 220 |

Cochrane Library

| ID | Search | Hits |
| --- | --- | --- |
| #1 | (cancer*):ti,ab,kw OR (carcinoma):ti,ab,kw OR (adenocarcinoma):ti,ab,kw OR (neoplas* ):ti,ab,kw OR (dysplas* ):ti,ab,kw OR (dyskaryos* ):ti,ab,kw OR (squamous):ti,ab,kw OR ( CIN ):ti,ab,kw OR (CINII* ):ti,ab,kw OR (CIN2* ):ti,ab,kw OR (CINIII* ):ti,ab,kw OR (CIN3* ):ti,ab,kw OR (SIL):ti,ab,kw OR (HSIL):ti,ab,kw OR (H-SIL):ti,ab,kw OR ( L-SIL):ti,ab,kw OR (LSIL):ti,ab,kw OR (ASCUS):ti,ab,kw OR (AS-CUS):ti,ab,kw | 244898 |
| #2 | MeSH descriptor: [Uterine Cervical Neoplasms] explode all trees | 2997 |
| #3 | (cervix):ti,ab,kw OR (cervical):ti,ab,kw OR (cervico*):ti,ab,kw (Word variations have been searched) | 29374 |
| #4 | #3 AND #1 | 8949 |
| #5 | #2 OR #4 | 8949 |
| #6 | MeSH descriptor: [Early Detection of Cancer] explode all trees | 2068 |
| #7 | (screen*):ti,ab,kw OR (screening):ti,ab,kw (Word variations have been searched) | 98853 |
| #8 | #6 OR #7 | 99078 |
| #9 | #5 AND #8 | 1981 |
| #10 | MeSH descriptor: [Human Papillomavirus DNA Tests] explode all trees | 11 |
| #11 | (Human papillomavirus test):ti,ab,kw OR (HPV DNA Test*):ti,ab,kw OR (Human Papillomavirus DNA Test*):ti,ab,kw OR (alphapapillomavirus):ti,ab,kw (Word variations have been searched) | 1447 |
| #12 | #10 OR #11 | 1447 |
| #13 | MeSH descriptor: [Papanicolaou Test] explode all trees | 334 |
| #14 | (Pap smear):ti,ab,kw OR (Papanicolaou smear):ti,ab,kw OR (Pap test):ti,ab,kw OR (Papanicolaou Test*):ti,ab,kw (Word variations have been searched) | 1985 |
| #15 | #13 OR #14 | 1985 |
| #16 | #9 OR #12 OR #15 | 4051 |
| #17 | (mobile phone):ti,ab,kw OR (smartphone):ti,ab,kw OR (text-message):ti,ab,kw OR (electronic-message):ti,ab,kw OR (multimedia-message):ti,ab,kw OR (electronic mail):ti,ab,kw OR (phone applications):ti,ab,kw OR (computer):ti,ab,kw OR (podcast):ti,ab,kw OR (videos):ti,ab,kw OR (internet):ti,ab,kw OR (website):ti,ab,kw OR (chatroom):ti,ab,kw OR (message board):ti,ab,kw OR (activity tracker):ti,ab,kw OR (electronic health):ti,ab,kw OR (mobile health):ti,ab,kw OR (telemedicine):ti,ab,kw OR (electronic learning):ti,ab,kw | 98312 |
| #18 | #16 AND #17 | 373 |

Embace

| #1 | cancer*':ab,ti OR 'carcinoma':ab,ti OR 'adenocarcinoma':ab,ti OR 'neoplas*':ab,ti OR 'dysplas*':ab,ti OR 'dyskaryos*':ab,ti | 4319913 |
| --- | --- | --- |
| #2 | uterine cervical neoplasms':ab,ti OR 'uterine cervical dysplasia':ab,ti OR 'cervical intraepithelial neoplasia':ab,ti | 11156 |
| #3 | 'cervix':ab,ti OR 'cervical':ab,ti OR 'cervico*':ab,ti | 404200 |
| #4 | #1 AND #3 | 173904 |
| #5 | #4 OR #2 | 404200 |
| #6 | 'human papillomavirus test':ab,ti OR 'hpv dna test*':ab,ti OR 'human papillomavirus dna test*':ab,ti OR 'alphapapillomavirus':ab,ti OR 'papanicolaou test':ab,ti OR 'pap smear':ab,ti OR 'papanicolaou smear':ab,ti | 11155 |
| #7 | screening':ab,ti OR 'screen*':ab,ti OR 'early detection of cancer':ab,ti | 1400625 |
| #8 | #5 AND #7 | 39038 |
| #9 | #6 OR #8 | 44748 |
| #10 | mobile phone':ab,ti OR 'smartphone':ab,ti OR 'text-message':ab,ti OR 'electronic-message':ab,ti OR 'multimedia-message':ab,ti OR 'electronic mail':ab,ti OR 'phone applications':ab,ti OR 'computer':ab,ti OR 'podcast':ab,ti OR 'videos':ab,ti OR 'internet':ab,ti OR 'website':ab,ti OR 'chatroom':ab,ti OR 'message board':ab,ti OR 'activity tracker':ab,ti OR 'electronic health':ab,ti OR 'mobile health':ab,ti OR 'telemedicine':ab,ti OR 'electronic learning':ab,ti | 568768 |
| #11 | #9 AND #10 | 1284 |
| #12 | 'rct':ab,ti OR 'randomized controlled trial':ab,ti | 185232 |
| #13 | #11 AND #12 | 32 |

CINAHL

| S12 | S10 AND S11 | 4 |
| --- | --- | --- |
| S11 | SU RCT OR SU Randomized controlled trial | 141454 |
| S10 | S7 AND S9 | 158 |
| S9 | S6 OR S8 | 6652 |
| S8 | SU Human papillomavirus test OR SU HPV DNA Test* OR SU Human Papillomavirus DNA Test* OR SU alphapapillomavirus OR SU Papanicolaou Test OR SU Pap smear OR SU Papanicolaou Smear OR SU Pap test | 6 |
| S7 | SU mobile phone OR SU smartphone OR SU text-message OR SU electronic-message OR SU multimedia-message OR SU electronic mail OR SU phone applications OR SU computer OR SU podcast OR SU videos OR SU internet OR SU website OR SU chatroom OR SU message board OR SU activity tracker OR SU electronic health OR SU mobile health OR SU telemedicine OR SU electronic learning | 239649 |
| S6 | S4 AND S5 | 6609 |
| S5 | SU Screening OR SU screen* OR SU Early Detection of Cancer | 104281 |
| S4 | S2 AND S3 | 21521 |
| S3 | SU cancer* OR SU carcinoma OR SU adenocarcinoma OR SU neoplas* OR SU dysplas* OR SU dyskaryos* OR SU squamous OR SU CIN OR SU CINII* OR SU CIN2* OR SU CINIII* OR SU CIN3* OR SU SIL OR SU HSIL OR SU H-SIL OR SU L-SIL OR SU LSIL OR SU ASCUS OR SU AS-CUS | 623397 |
| S2 | SU cervix OR SU cervical OR SU cervico* | 42408 |
| S1 | SU Uterine Cervical Neoplasms OR SU Uterine Cervical Dysplasia OR SU Cervical Intraepithelial Neoplasia | 2821 |

Scopus

( TITLE-ABS ( rct ) OR TITLE-ABS ( "Randomized controlled trial" ) ) AND ( ( TITLE-ABS ( "mobile phone" ) OR TITLE-ABS ( "smartphone" ) OR TITLE-ABS ( "text-message" ) OR TITLE-ABS ( "electronic-message" ) OR TITLE-ABS ( "multimedia-message" ) OR TITLE-ABS ( "electronic mail" ) OR TITLE-ABS ( "phone applications" ) OR TITLE-ABS ( "computer" ) OR TITLE-ABS ( "podcast" ) OR TITLE-ABS ( "videos" ) OR TITLE-ABS ( "internet" ) OR TITLE-ABS ( "website" ) OR TITLE-ABS ( "chatroom" ) OR TITLE-ABS ( "message board" ) OR TITLE-ABS ( "activity tracker" ) OR TITLE-ABS ( "electronic health" ) OR TITLE-ABS ( "mobile health" ) OR TITLE-ABS ( "telemedicine" ) OR TITLE-ABS ( "electronic learning" ) ) AND ( ( ( TITLE-ABS ( "Early Detection of Cancer" ) OR TITLE-ABS ( screening ) OR TITLE-ABS ( screen* ) ) AND ( ( TITLE-ABS ( cancer* ) OR TITLE-ABS ( carcinoma ) OR TITLE-ABS ( adenocarcinoma ) OR TITLE-ABS ( neoplas* ) OR TITLE-ABS ( dysplas* ) OR TITLE-ABS ( dyskaryos* ) OR TITLE-ABS ( squamous ) OR TITLE-ABS ( cin ) OR TITLE-ABS ( cinii* ) OR TITLE-ABS ( cin2* ) OR TITLE-ABS ( ciniii* ) OR TITLE-ABS ( cin3* ) OR TITLE-ABS ( sil ) OR TITLE-ABS ( hsil ) OR TITLE-ABS ( h-sil ) OR TITLE-ABS ( l-sil ) OR TITLE-ABS ( lsil ) OR TITLE-ABS ( ascus ) OR TITLE-ABS ( as-cus ) ) AND ( TITLE-ABS ( cervix ) OR TITLE-ABS ( cervical ) OR TITLE-ABS ( cervico* ) ) ) ) OR ( TITLE-ABS ( human AND papillomavirus AND test ) OR TITLE-ABS ( hpv AND dna AND test* ) OR TITLE-ABS ( human AND papillomavirus AND dna AND test* ) OR TITLE-ABS ( alphapapillomavirus ) OR TITLE-ABS ( papanicolaou AND test ) OR TITLE-ABS ( pap AND smear ) OR TITLE-ABS ( papanicolaou AND smear ) OR TITLE-ABS ( pap AND test ) ) ) )

MEDLIN

| S11 | S5 AND S10 | 7 |
| --- | --- | --- |
| S10 | S4 AND S9 | 448 |
| S9 | S2 OR S8 | 14203 |
| S8 | S3 AND S7 | 8741 |
| S7 | S1 AND S6 | 51440 |
| S6 | SU cervix OR SU cervical OR SU cervico* | 90058 |
| S5 | SU RCT OR SU Randomized controlled trial | 115702 |
| S4 | SU mobile phone OR SU smartphone OR SU text-message OR SU electronic-message OR SU multimedia-message OR SU electronic mail OR SU phone applications OR SU computer OR SU podcast OR SU videos OR SU internet OR SU website OR SU chatroom OR SU message board OR SU activity tracker OR SU electronic health OR SU mobile health OR SU telemedicine OR SU electronic learning | 563990 |
| S3 | SU Screening OR SU screen* OR SU Early Detection of Cancer | 171667 |
| S2 | SU Human papillomavirus test OR SU HPV DNA Test* OR SU Human Papillomavirus DNA Test* OR SU alphapapillomavirus OR SU Papanicolaou Test OR SU Pap smear OR SU Papanicolaou Smear OR SU Pap test | 7538 |
| S1 | SU cancer* OR SU cancer* OR SU carcinoma OR SU adenocarcinoma OR SU neoplas* OR SU dysplas* OR SU dyskaryos* OR SU squamous OR SU CIN OR SU CINII* OR SU CIN2* OR SU CINIII* OR SU CIN3* OR SU SIL OR SU HSIL OR SU H-SIL OR SU L-SIL OR SU LSIL OR SU ASCUS OR SU AS-CUS OR | 1913393 |

APA PsycInfo


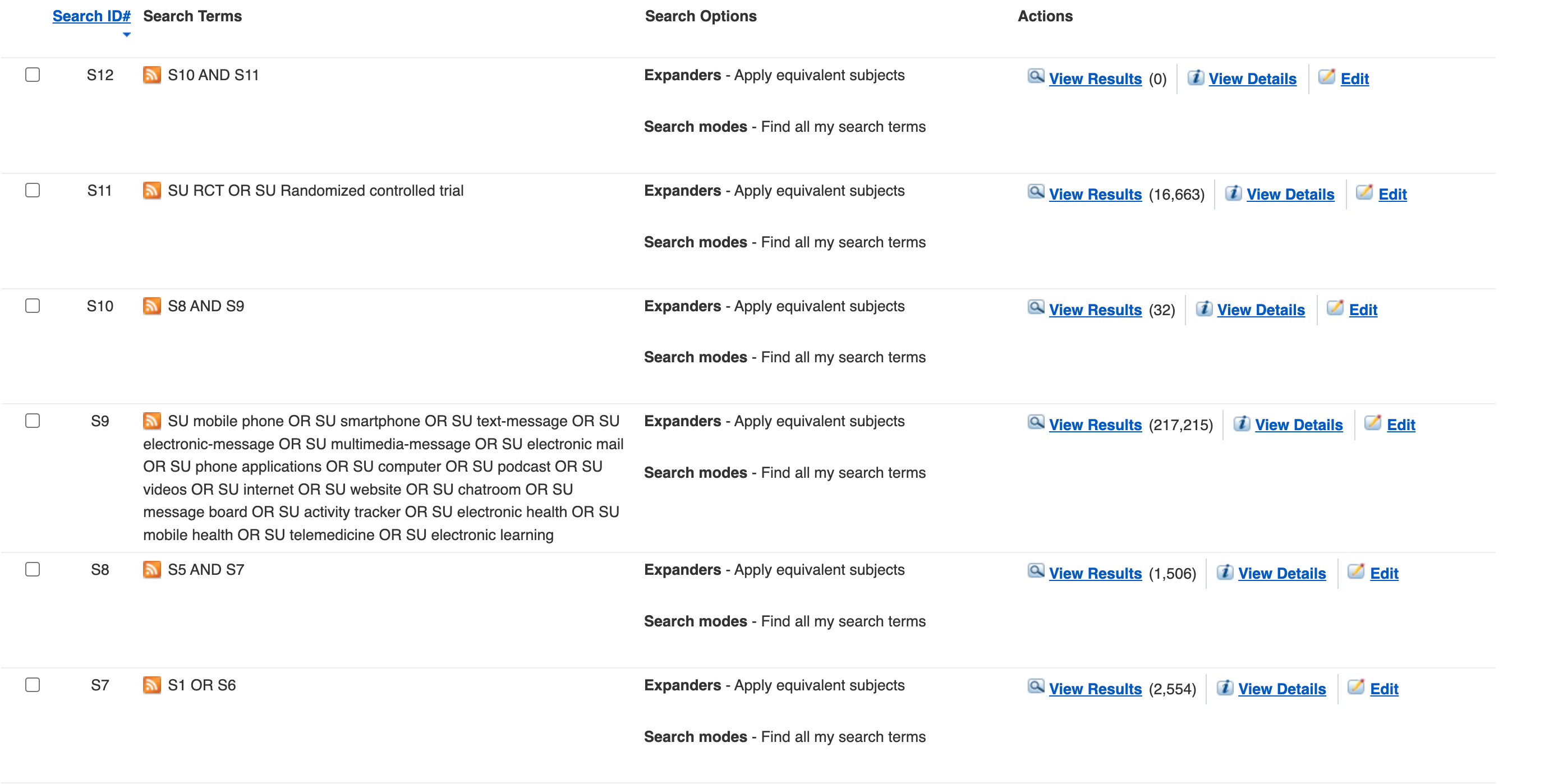

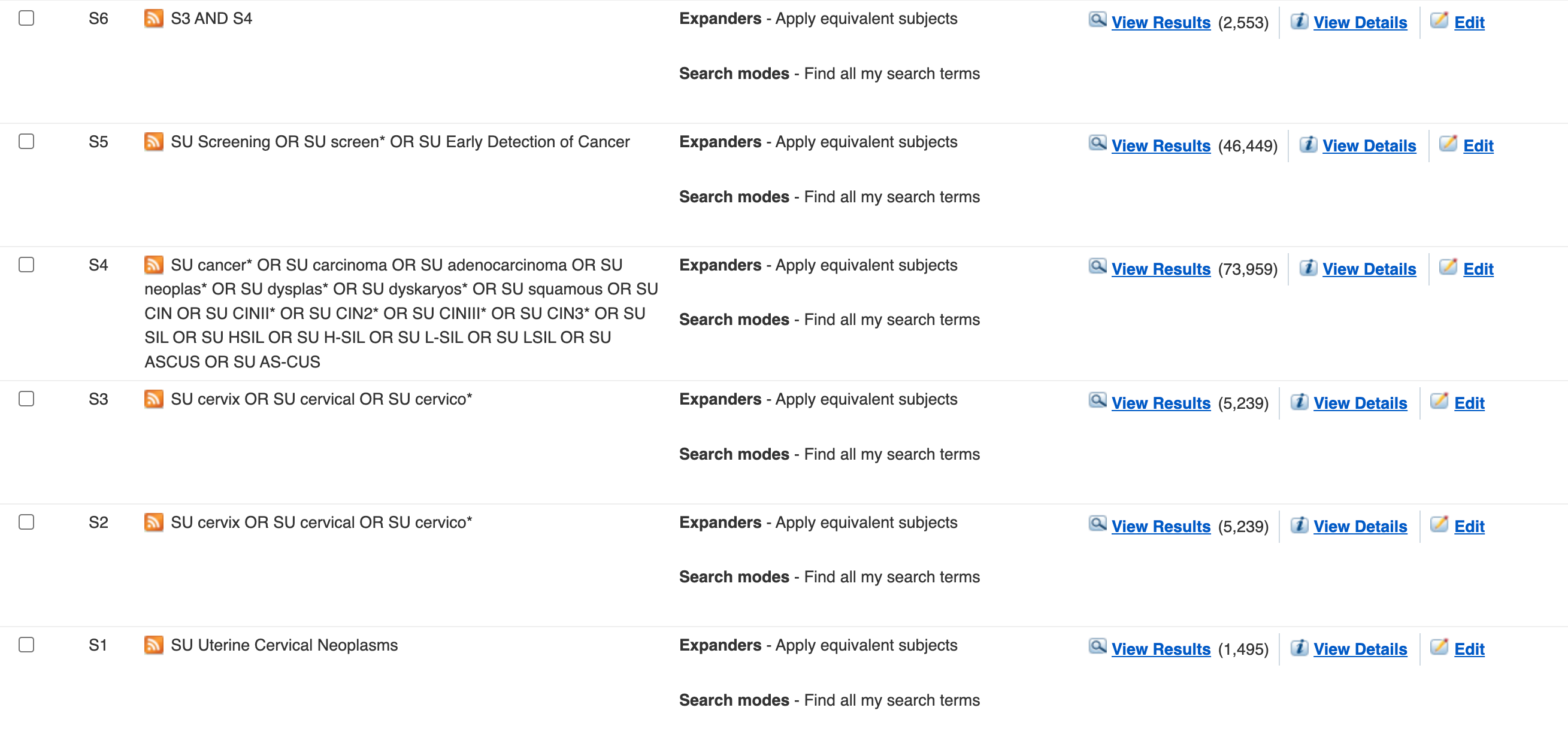

Supplement: Multimedia Appendix 2 [file jmir_v26i1e58066_app2.docx]
