# Supplementary material for: Electronic Health Interventions and Cervical Cancer Screening: Systematic Review and Meta-Analysis
Source: J Med Internet Res. 2024 Oct 31;26:e58066. doi: 10.2196/58066 (PMC11565089; doi:10.2196/58066)

Supplement Table 1. Summary of included randomized controlled trials (n=14).

| ID | Author,  Year,  Country,  Citation | List  intervention  groups | Most complex eHealth  intervention details | Comparator | Population  (most complex  intervention n/control n;years old) | Intervention  duration;  follow-up (mos) | Primary  outcome | Secondary outcomes | Applying model |
| --- | --- | --- | --- | --- | --- | --- | --- | --- | --- |
|  |  |  |  |  |  |  |  |  |  |
| 1 | Fauziah Abdullah 2013 Malaysia | phone call | a personal invitation letter with an information pamphlet of cervical cancer screening, and followed by a telephone reminder with counseling after four weeks that was performed once per participant. | usual care | teacher(201/202;36.3±8.1) | 6;6 | had a Pap test | N | the Transtheoretical Model (TTM) |
| 2 | David Adler 2019 American | referral and an SMS-based behavioral intervention aimed | participants in the intervention condition received a total of three text messages delivered at 30-day intervals over a period of 90 days after enrollment | referral | emergency(48/47;37.9±12.6) | 2;5 | CC screening | regarding barriers to care and perceptions of the study interventions. | Theory of Planned Behavior(TPB) |
| 3 | Silvina Arrossi 2022 Argentina | mhealth | HPV-positive women received one weekly SMS message over a four-week period, notifying that HPV results were available and that they should attend the health centre. | usual care | HPV-positive women(445/292;42.4±11.0) | 2;4 | CC screening | N | Health Belief Model( HBM) |
| 4 | Theresa L. Byrd 2013 American | AMIGAS(video) | A video that uses role modeling by women from the community to address common barriers and beliefs about cervical cancer and screening | usual care | Mexican American Women(155/153;N) | 6;6 | CC screening | N | Theoretical constructs from Social Cognitive Theory, the Health Belief Model, the Transtheoretical Model,and the Theory of Reasoned Action |
| 5 | Erica Erwin 2019 Tanzania | SMS | Three SMS were sent at enrolment and one SMS sent every 1 or 2 days thereafter until day 21 | usual care | women(272/281;34.4±7.2) | 2;2 | CC screening | N | the Health Belief Model |
| 6 | João Firmino-Machado 2019 Portugal | text messages, phone calls and reminders | 1:an invitation to cervical cancer screening through automated and customized text messages and phone calls; 2:this comprised an invitation through a phone call performed to Women remaining non-adherent up to 45days; 3:Women who remained non-adherent up to 45 days after step 2 invitations,This comprised a phone call and a face-to-face interview. | cervical cancer screening through a written letter | women(605/615;34.6±7.7) | 3;5 | CC screening | N | N |
| 7(1) | Sarah Huf 2020 England | SMS | Send a text message to the patient informing them of the screening and provide a screening appointment number | usual care | young women aged 20 to 29 (1539/1594;26.1±1.9) | 2;4.5 | CC screening | N | MINDSPACE framework |
| 7(2) | Sarah Huf 2020 England | SMS | Send a text message: “Failing to attend cervical screening could lead to 4500 avoidable deaths in England each year. Your cervical smear test is due. To book please call <GP phone number>” | usual care | women aged 30 to 64 (1611/1675;  42.9±9.3) | 2;4.6 | CC screening | N | MINDSPACE framework |
| 8 | Aslı Karakuş Selçuk 2019 Turkey | Web-based education | The research link was sent to the teachers via e-mail or WhatsApp. The educational video about CC and the PST was prepared professionally according to literature and with the recommendations of the experts. | usual care | teacher(678/612;37.1±7.9) | 1;3 | Cervical cancer knowledge | CC screening | N |
| 9 | H Kitchener 2018 England | Online booking | Provide screening booking links for women | usual care | young women(5267/4467;N) | 6;6 | CC screening | HPV vaccination | N |
| 10 | Ditte S Linde 2020 Tanzanian | One-Way Text Messaging | Over a period of 10 months, 10 health educative messages, and 5 reminders were sent to the women in the intervention group. The health educative messages were sent once a month. | usual care | HPV positive(358/347;N) | 10;14 | CC screening | cost-effectiveness of the intervention, the intervention’s effect on the knowledge of cervical cancer and screening | N |
| 11 | Zaahirah Mohammad 2022 Malaysia | Whatsapp | The role of the WhatsApp group was to share information, concerns, and issues; as well as address any misunderstanding on Pap smear and cervical cancer. Besides, it acts as a reminder. | usual care | women(201/200;N) | 1;3 | CC screening | knowledge, attitude, and self-efficacy | Social Cognitive Theory (SCT) |
| 12 | Beti Thompson 2017 Spain | web video | The video contained information about cervical cancer screening, encouragement to undergo screening, and information about low cost clinics where women could go for the screening. | usual care | Women(150/147;43.9±9.3) | N;7 | CC screening | change in knowledge and attitudes about cervical cancer risk factors and Pap testing | Social Cognitive Theory (SCT). |
| 13 | S. M. Peitzmeier 2016 Spain | multimodal outreach | 1: attempt consisted of the patients receiving a letter andeducational flyer as outlined for the letter outreach above. 2: consisted of the patients receiving an email with the educational attachment as outlined for the email outreach above. 3: consisted of telephone outreach as outlined for the telephone group above. If a mailing address or an email address was not available, then a telephone call was made to the patient instead. | usual care | Women who have not been screened for a long time (220/220;30.1) | 1.5;12 | CC screening | N | N |
| 14 | Rima Marhayu Abdul Rashid 2013 Malaysia | phone call | They were supposed to repeat the screening, the list of clinics that they can go to and phone numbers that they can call to re-schedule appointment if they necessary. | usual care | Women who had Pap smear in the previous year and is due for repeat screening(250/250;N) | N;2 | Respond to intervention | CC screening | N |

^N: Not reported in the included studies; CC: Cervical cancer; SMS: Short Messaging Service^

Supplement Table 2. The methodological quality of 14 articles was assessed using the Randomized Controlled Trial [Checklist](https://www.sciencedirect.com/topics/nursing-and-health-professions/checklist) of the Joanna Briggs Institute. The assessment criteria were marked as “Y” for “Yes”, “N” for “No”, and “U” for “Unclear”.

| ID | Author | year | -1 | -2 | -3 | -4 | -5 | -6 | -7 | -8 | -9 | -10 | -11 | -12 | -13 | Overall |
| --- | --- | --- | --- | --- | --- | --- | --- | --- | --- | --- | --- | --- | --- | --- | --- | --- |
| 1 | Fauziah Abd ullah | 2013 | Y | U | Y | U | U | Y | U | Y | Y | Y | Y | Y | Y | 9 |
| 2 | David Adler | 2019 | Y | Y | Y | U | U | Y | U | Y | U | U | N | Y | Y | 7 |
| 3 | Silvina Arrossi | 2022 | Y | Y | Y | Y | N | Y | N | Y | U | Y | Y | Y | Y | 10 |
| 4 | Theresa L. Byrd | 2013 | Y | U | Y | U | U | Y | Y | Y | Y | Y | Y | Y | Y | 10 |
| 5 | Erica Erwin | 2019 | Y | Y | N | Y | Y | Y | Y | Y | Y | Y | N | Y | Y | 11 |
| 6 | João Firmino-Machado | 2019 | Y | Y | Y | N | N | Y | N | Y | Y | Y | Y | Y | Y | 10 |
| 7 | Sarah Huf | 2020 | Y | Y | Y | N | N | Y | N | Y | Y | Y | Y | Y | Y | 10 |
| 8 | Aslı Karakuş Selçuk | 2019 | N | N | Y | U | U | Y | U | Y | U | Y | N | Y | U | 5 |
| 9 | H Kitchener | 2018 | Y | Y | U | U | U | Y | U | Y | Y | Y | Y | Y | Y | 9 |
| 10 | Ditte S Linde | 2020 | Y | Y | Y | N | Y | Y | Y | Y | Y | Y | Y | Y | Y | 12 |
| 11 | Zaahirah Mohammad | 2022 | Y | Y | Y | Y | N | Y | Y | Y | U | Y | Y | Y | Y | 11 |
| 12 | Beti Thompson | 2017 | Y | Y | Y | Y | Y | Y | Y | Y | Y | Y | Y | Y | Y | 13 |
| 13 | S. M. Peitzmeier | 2018 | Y | Y | Y | U | U | Y | U | Y | Y | Y | Y | Y | U | 9 |
| 14 | Rima Marhayu Abdul Rashid | 2013 | Y | Y | Y | Y | Y | Y | Y | Y | U | Y | Y | Y | Y | 12 |

Items: 1. Was true randomization used for assignment of participants to treatment groups? 2. Was allocation to treatment groups concealed? 3. Were treatment groups similar at the baseline? 4. Were participants blind to treatment assignment? 5. Were those delivering treatment blind to treatment assignment? 6. Were outcomes assessors blind to treatment assignment? 7. Were treatment groups treated identically other than the intervention of interest? 8. Was follow-up complete and if not, were differences between groups in terms of their follow-up adequately described and analyzed? 9. Were participants analyzed in the groups to which they were randomized? 10. Were outcomes measured in the same way for treatment groups? 11. Were outcomes measured in a reliable way? 12. Was appropriate statistical analysis used? 13. Was the trial design appropriate, and any deviations from the standard RCT design (individual randomization, parallel groups) accounted for in the conduct and analysis of the trial?

Supplement Figure 1: Funnel plot comparing electronic health interventions to non-electronic health interventions in randomized controlled trials reporting on intention-to-treat analysis of cervical cancer screening. RR: relative risk.


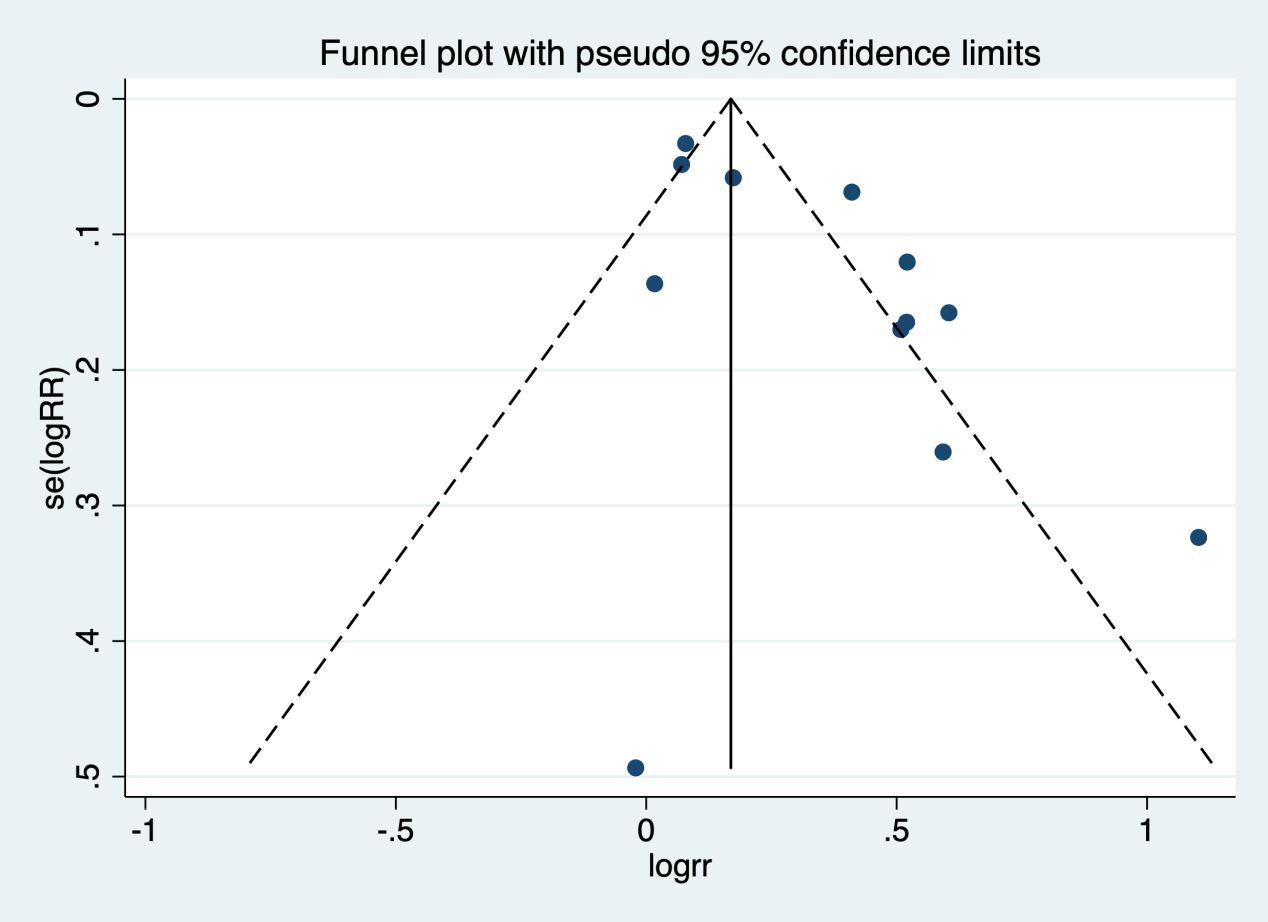


Egger's test: t: 3.09, P: 0.011, 95%CI: 0.71 - 4.01

Table 3 The result of Duval and Tweedie trim-and-fill analysis

| Method | Est | Lower | Upper | z_value | p_value | studies |
| --- | --- | --- | --- | --- | --- | --- |
| Fixed | 0.167 | 0.592 | 0.926 | 0.431 | 0.666 | 12 |
| Random \| | 0.167 | 1.592 | 1.926 | 1.431 | 1.666 | -- |

Test for heterogeneity: Q= 0.158 on 11 degrees of freedom (p= 1.000)

Moment-based estimate of between studies variance = 0.000

Note: no trimming performed; data unchanged

Supplement Figure 2: Funnel plot comparing electronic health interventions to non-electronic health interventions in randomized controlled trials reporting on per-protocol analysis of cervical cancer screening. RR: relative risk.


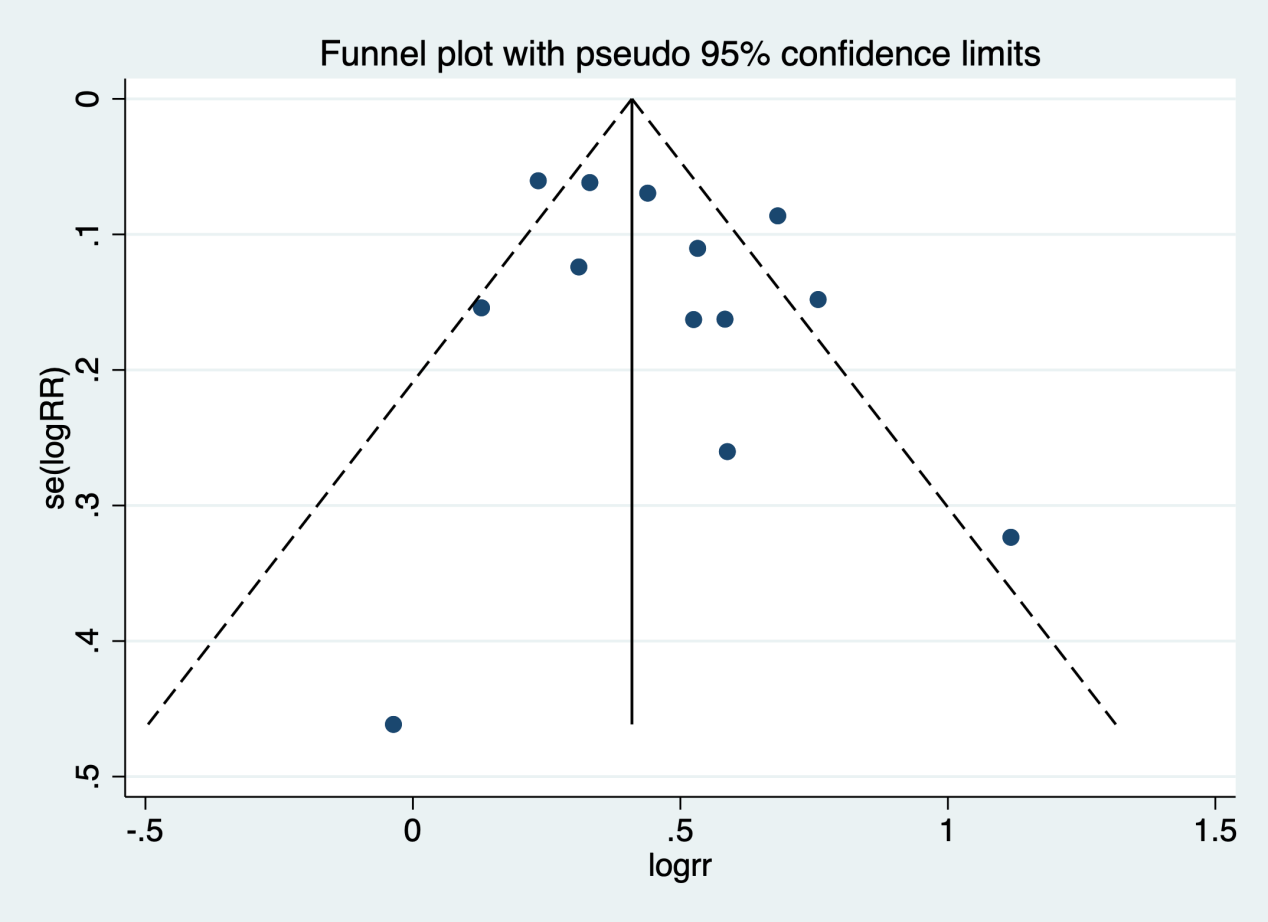


Egger's test: t:1.31, P:0.217, 95%CI: -0.94,3.55

Supplement Figure 3: Forest plot comparing electronic health interventions to non-electronic health interventions in randomized controlled trials for cervical cancer screening participation by intervention type. RR: relative risk.


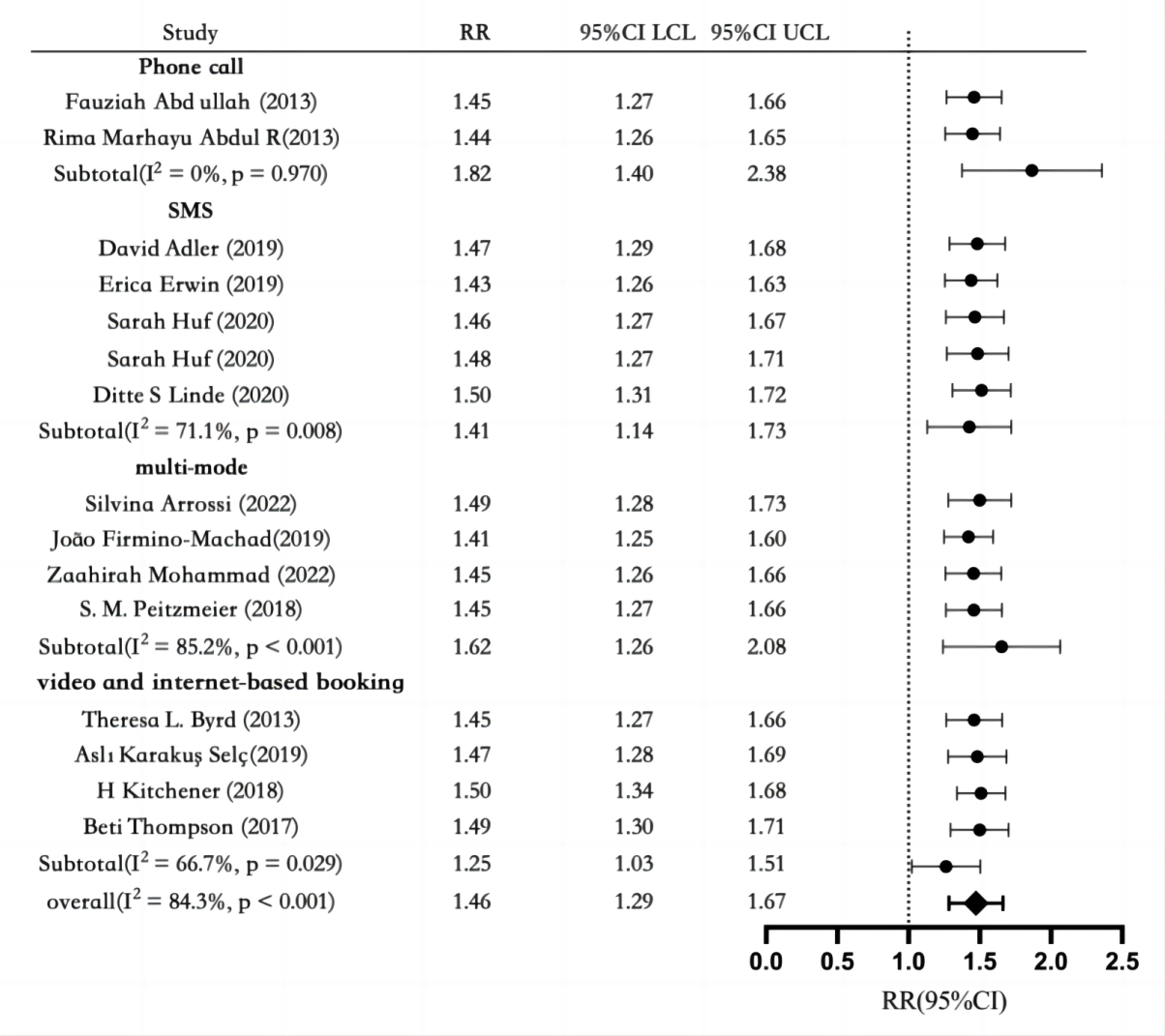


1 phone call: Tau^2^ = 0.0000,chi^2^ = 0.0000,P = 0.970, I^2^ = 0.0%, Z = 4.45, P < 0.00001

2 SMS: Tau^2^ = 0.0303, chi^2^ = 13.82, P = 0.008,I^2^ = 71.1%, Z = 3.22. P = 0.001

3 muiti-mode: Tau^2^ = 0.0536, chi^2^ = 20.22, P = 0.000,I^2^ = 85.2%, Z = 3.76. P < 0.00001

4 video and internet-net booking: Tau^2^ = 0.0450, chi^2^ = 9.00, P = 0.029,I^2^ = 66.7%, Z = 2.29, P =0.022

Supplement Figure 4: Forest plot comparing electronic health interventions to non-electronic health interventions in randomized controlled trials for cervical cancer screening participation by subject. RR: relative risk.


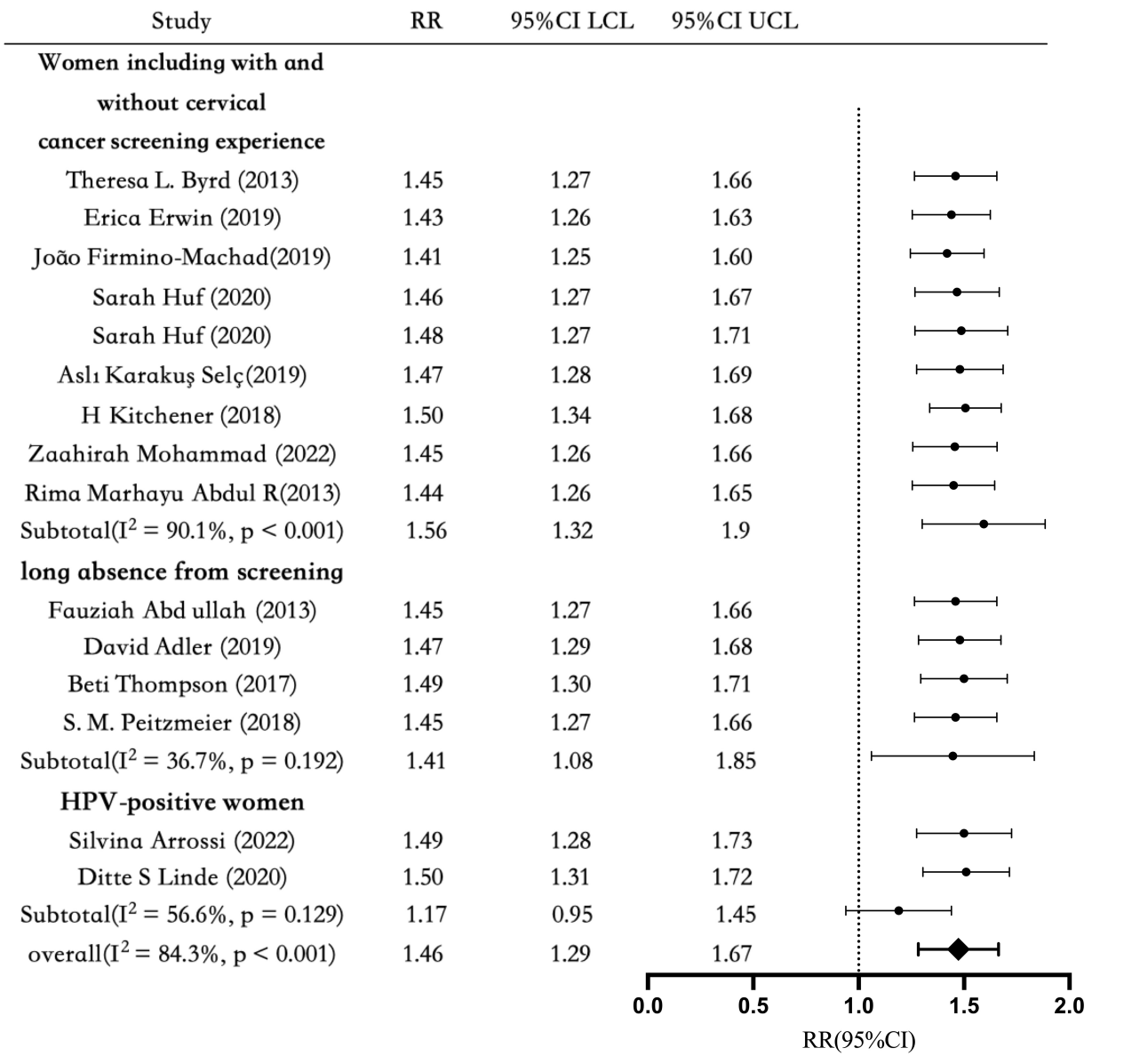


1 Women include with and without cervical cancer screening experience: Tau^2^ = 0.0622,chi^2^ = 4.74,P = 0.000, I^2^ = 90.1%, Z = 4.91, P < 0.00001

2 Long time ansence from screening: Tau^2^ = 0.0271, chi^2^ = 4.74, P = 0.192, I^2^ = 36.7%, Z = 2.50. P = 0.012

3 HPV positivity: Tau^2^ = 0.0145, chi^2^ = 2.30, P = 0.129, I^2^ = 56.6%, Z = 1.45. P = 0.146

Supplement Figure 5: Forest plot comparing electronic health interventions to non-electronic health interventions in randomized controlled trials for cervical cancer screening participation by economic level. RR: relative risk.


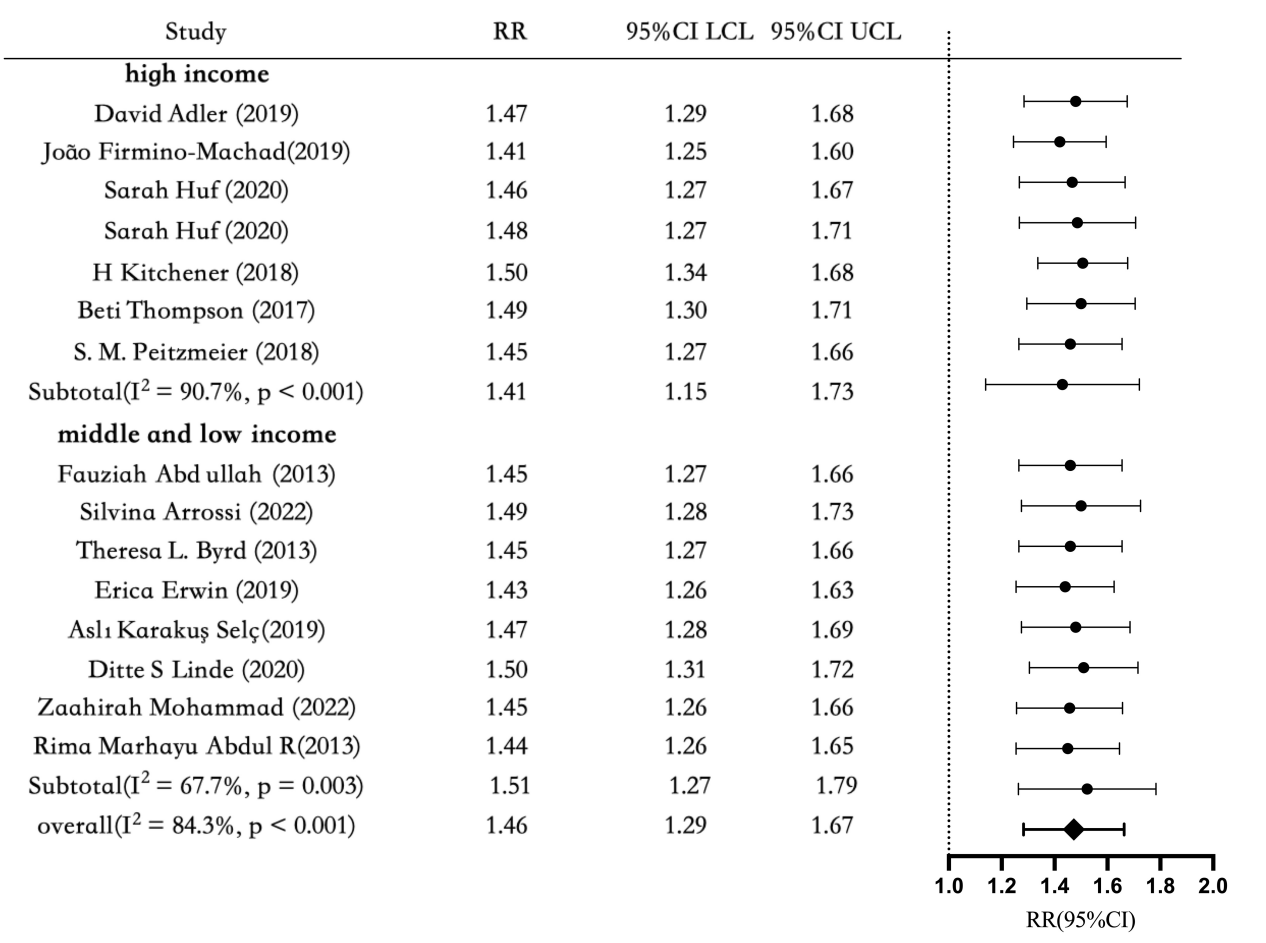


1 High income economy: Tau^2^ = 0.0588, chi^2^ = 64.68,P = 0.000, I^2^ = 90.7%, Z = 3.28, P = 0.001

2 Middle and low income economy: Tau^2^ = 0.0367, chi^2^ = 64.68, P = 0.003, I^2^ = 67.7%, Z = 4.68. P < 0.00001

Supplement Figure 6: Sensitivity analysis comparing electronic health interventions to non-electronic health interventions for the cervical cancer screening participation influence of each study on the pooled estimates. The leave-one-out approach was used (n = 14).


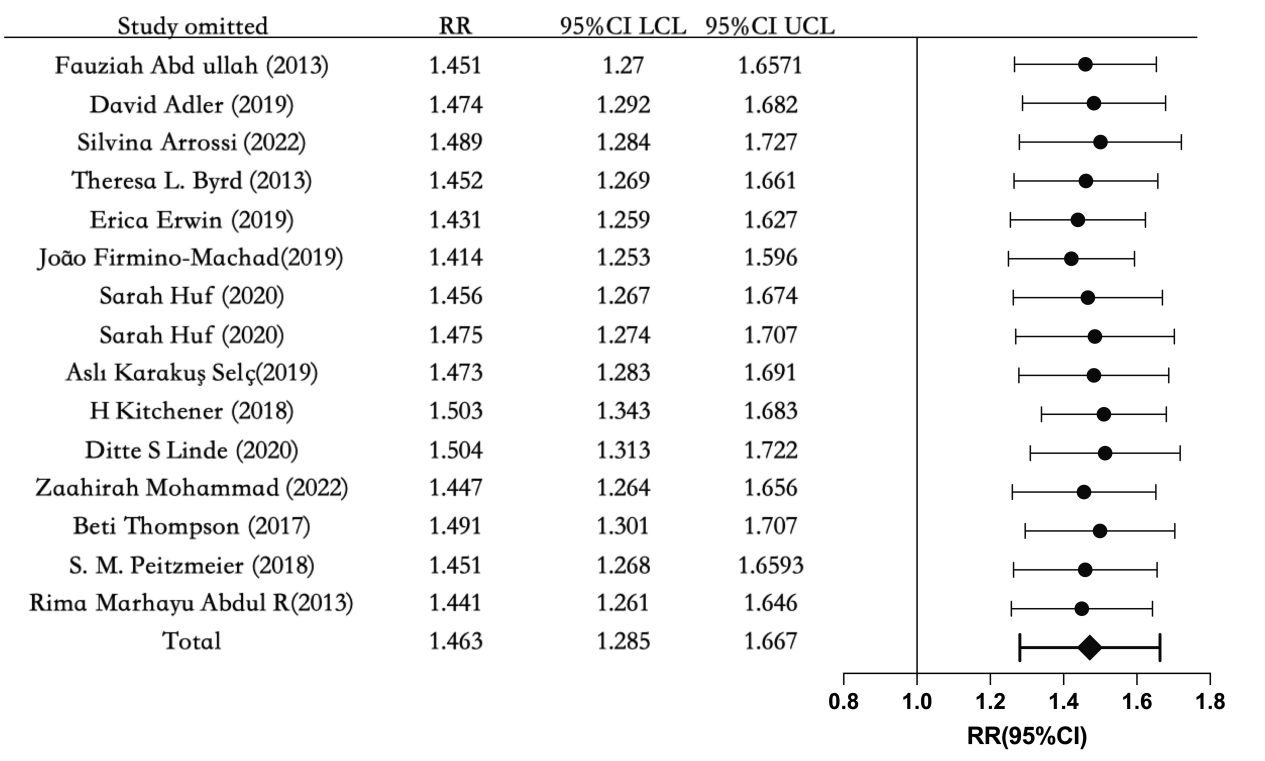


Supplement Figure 7: Sensitivity analysis comparing electronic health interventions to non-electronic health interventions for the cervical cancer screening participation inclusion of randomized controlled trials of excluding studies with high risk of bias (n = 7).


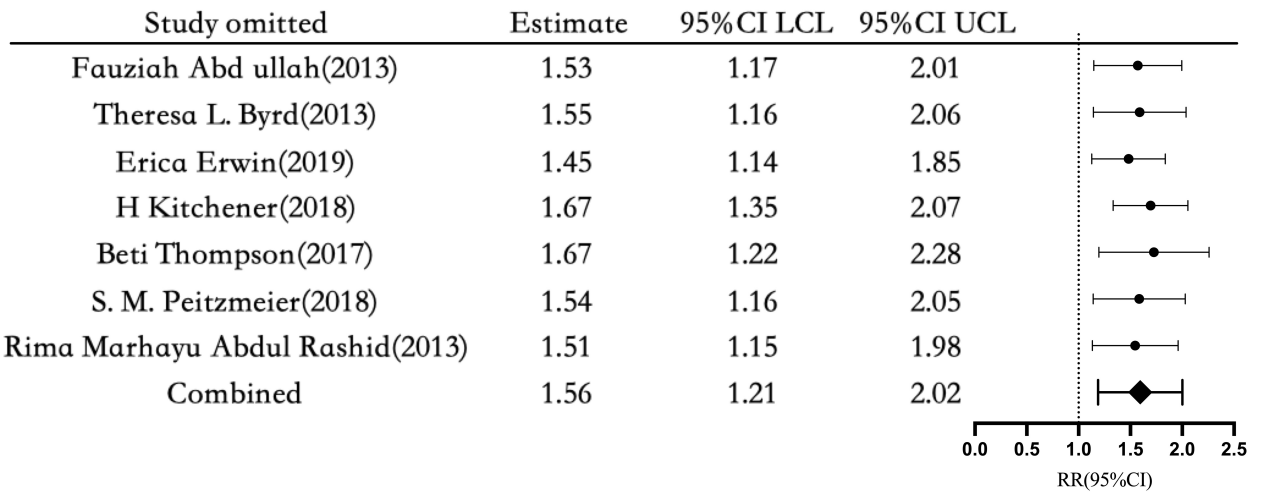


Supplement Figure 8: Sensitivity analysis comparing electronic health interventions to non-electronic health interventions for the intention-to-treat analysis of cervical cancer screening participation influence of each study on the pooled estimates. The leave-one-out approach was used (n = 11).


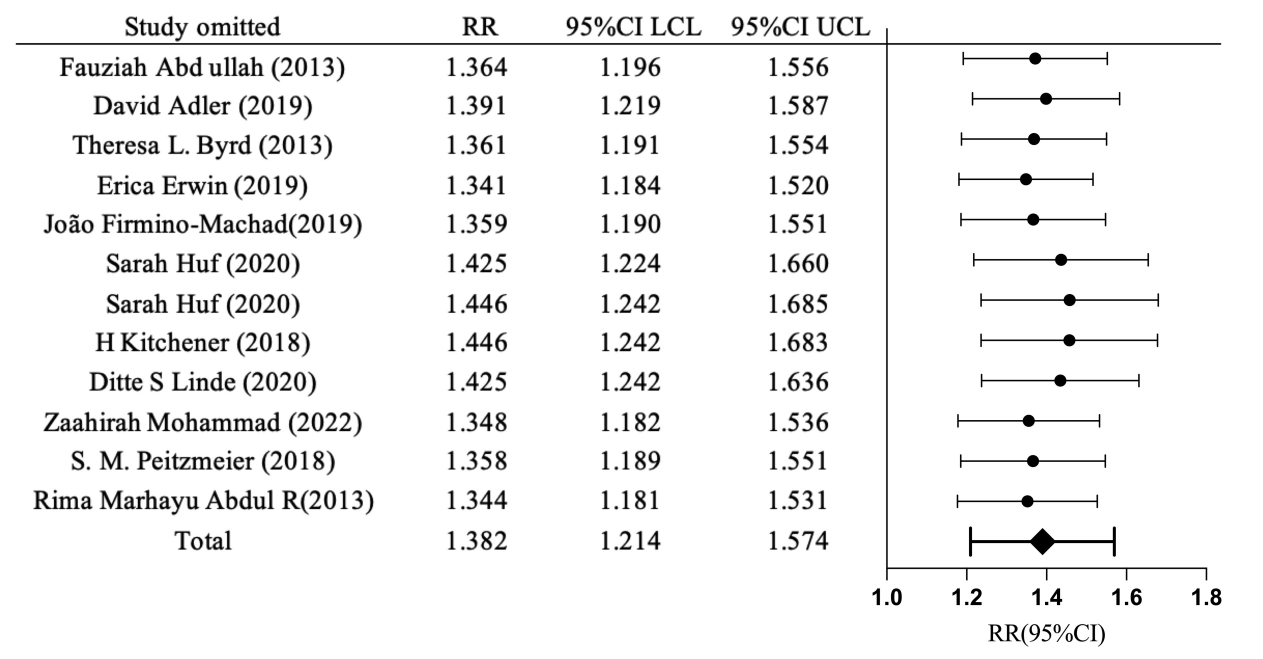


Supplement Figure 9: Sensitivity analysis comparing electronic health interventions to non-electronic health interventions for the intention-to-treat analysis of cervical cancer screening participation inclusion of randomized controlled trials of excluding studies with high risk of bias (n=6).


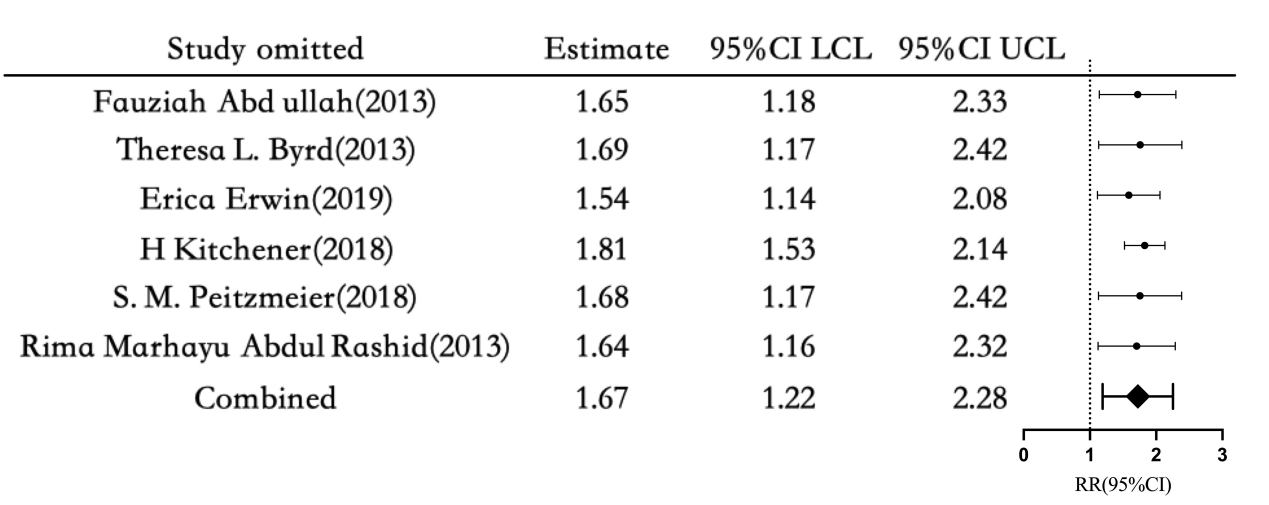


Supplement Figure 10: Sensitivity analysis comparing electronic health interventions to non-electronic health interventions for the per-protocol analysis of cervical cancer screening participation influence of each study on the pooled estimates. The leave-one-out approach was used (n=12).


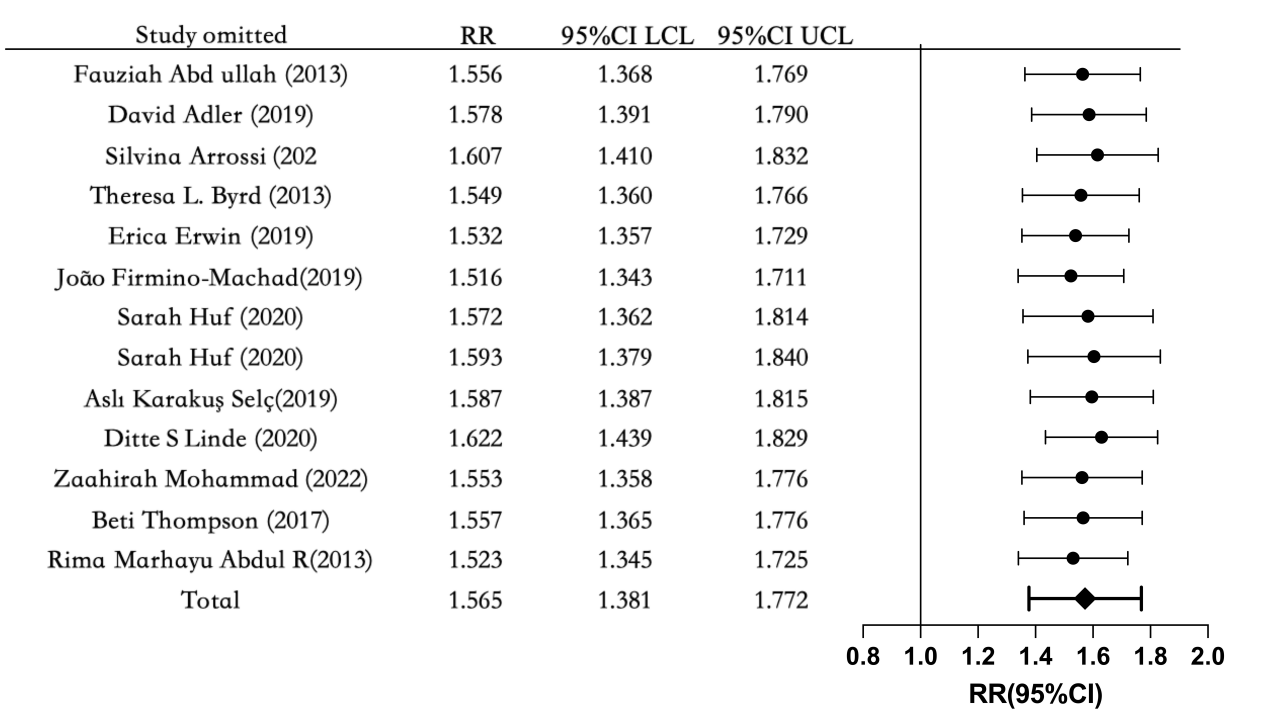


Supplement Figure 11: Sensitivity analysis comparing electronic health interventions to non-electronic health interventions for the per-protocol analysis of cervical cancer screening participation inclusion of randomized controlled trials of excluding studies with high risk of bias (n = 5).


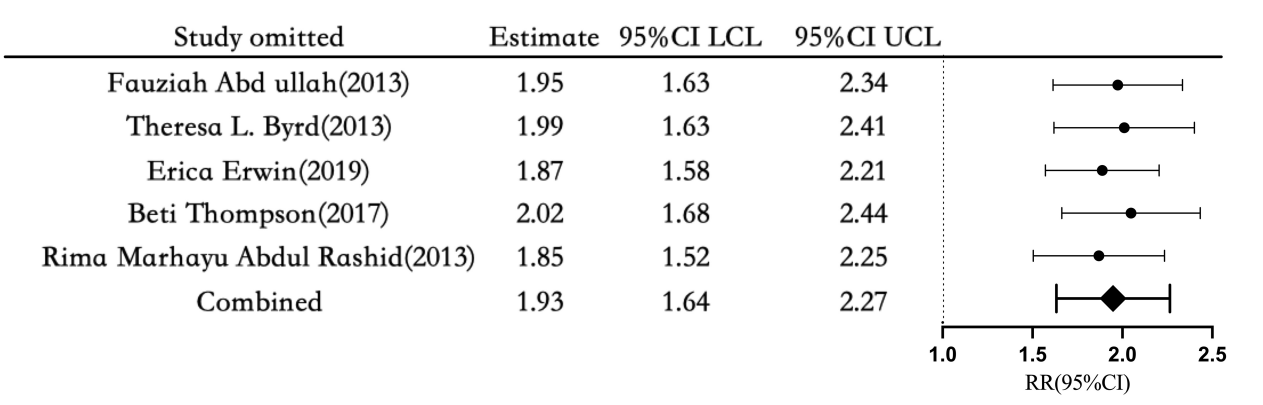

Supplement: Multimedia Appendix 3 [file jmir_v26i1e58066_app3.docx]
